# A four‐factor immune risk score signature predicts the clinical outcome of patients with spinal chordoma

**DOI:** 10.1002/ctm2.4

**Published:** 2020-05-13

**Authors:** Ming‐Xiang Zou, Yue Pan, Wei Huang, Tao‐Lan Zhang, David Escobar, Xiao‐Bin Wang, Yi Jiang, Xiao‐Ling She, Guo‐Hua Lv, Jing Li

**Affiliations:** ^1^ Department of Spine Surgery The First Affiliated Hospital University of South China Hengyang China; ^2^ Department of Spine Surgery The Second Xiangya Hospital Central South University Changsha China; ^3^ Institute of Precision Medicine Xiangya Hospital Central South University Changsha China; ^4^ Department of Cancer Biology College of Medicine & Life Sciences University of Toledo Toledo Ohio; ^5^ Department of Pathology The Second Xiangya Hospital Central South University Changsha China

**Keywords:** immune risk score, multiplex immunofluorescence, nomogram, spinal chordoma, tumor‐infiltrating lymphocytes

## Abstract

**Background:**

Currently, the measurement of immune cells in previous studies is usually subjective, and no immune‐based prognostic model has been established for chordoma. In this study, we sought to simultaneously measure tumor‐infiltrating lymphocyte (TIL) subtypes in chordoma samples using an objective method and develop an immune risk score (IRS) model for survival prediction.

**Methods:**

Multiplexed quantitative immunofluorescence staining was used to determine the TIL levels in the tumoral and stromal subareas of 114 spinal chordoma specimens (54 in the training and 60 in the validation cohort) for programmed death‐1 (PD‐1), CD3, CD8, CD20 (where CD is cluster of differentiation), and FOXP3. Flow cytometry was performed to validate the immunofluorescence assay for lymphocyte measurement on an additional five fresh chordoma specimens. Subsequently, the IRS model was built using the least absolute shrinkage and selection operator (LASSO) Cox regression method.

**Results:**

Flow cytometry and quantitative immunofluorescence showed similar lymphocytic percentages and TIL subpopulation proportions in the fresh tumor specimens. With the training data, the LASSO model identified four immune features for IRS construction: _tumoral_FOXP3, _tumoral_PD‐1, _stromal_FOXP3, and _stromal_CD8. In both cohorts, a high IRS was significantly associated with tumoral programmed cell death‐1 ligand 1 expression, Enneking inappropriate tumor resection, and surrounding muscle invasion by tumor. Multivariate Cox regression and stratified analysis in the two cohorts revealed that the IRS was an independent predictor and could effectively separate patients with similar Enneking staging into different risk subgroups, with significantly different survival rates. Further receiver operating characteristic analysis found that the IRS classifier had a better prognostic value than the traditional clinicopathological factors and compensated for the deficiency of Enneking staging for outcome prediction. More importantly, a nomogram based on the IRS and clinical predictors showed adequate performance in estimating disease recurrence and survival of patients.

**Conclusions:**

These data support the use of the IRS signature as a reliable prognostic tool in spinal chordoma and may facilitate individualized therapy decision making for patients.

**Abbreviations**
TIL: tumor‐infiltrating lymphocyte
IRS: immune risk score
H&E: hematoxylin and eosin
FFPE: formalin‐fixed paraffin‐embedded
LRFS: local relapse‐free survival
OS: overall survival
AOI: areas of interest
DAPI: 4’,6‐diamidino‐2‐phenylindole
LASSO: least absolute shrinkage and selection operator
ROC: receiver operating characteristic
EA: Enneking appropriate
EI: Enneking inappropriate
APC: allophycocyanin
PD‐1: programmed death‐1
PD‐L1: programmed cell death‐1 ligand 1
CD: cluster of differentiation

## 1 BACKGROUND

Chordoma is a rare mesenchymal malignancy that shows slow growth and is considered to originate from notochord remnants.^1^ Chordoma has an incidence rate of approximately 0.18‐0.84 per million persons each year and most commonly involves the axial skeleton.^2^ Clinically, chordoma is resistant to traditional chemotherapy or radiotherapy,^1^, ^3^ and thus, complete surgical excision is the most effective treatment for this disease.^4^ However, radical *en bloc* resection of these tumors can be technically demanding due to their infiltrative nature and proximity to vital neurovascular structures.^5^ Therefore, patients are vulnerable to recurrence after surgery, and 40‐50% of them can even develop metastasis.^6^ Given the dismal prognosis of patients with chordoma, exploring improved treatment strategies is urgently needed at present.

The tumor microenvironment represents an integral part of cancer^7^ and is composed of cancer cells, stromal cells, extracellular matrix, and various nonmalignant host cells, especially immune cells.^7^, ^8^ Recently, studies have suggested a key role for microenvironmental immune cells in prognostic risk stratification^9^, ^10^ and selection of cancer patients who can undergo immunotherapy.^10^, ^11^, ^12^ For example, researchers have found that tumor‐infiltrating lymphocytes (TILs) in the tumor microenvironment are reliable predictors of the clinical outcome of human cancers.^9^ In addition, it has been demonstrated that immune cell infiltrates, which represent the preexisting immunity of tumors, are closely associated with the drug response to immune checkpoint blockade therapy.^10^, ^12^, ^13^, ^14^ However, the measurement of TILs in most studies using hematoxylin and eosin (H&E)‐based pathologist estimation or single‐color immunohistochemical technology is semiquantitative and subjective. Although automated quantification has been currently proposed to evaluate TILs,^15^, ^16^ this method has a limited ability for multiple cellular subtyping in a compartment‐specific manner.^17^ Flow cytometry, which fails to capture architectural information despite its ability to simultaneously analyze multiple parameters, is similarly limited. Considering these issues, researchers have now begun to employ multiplexed quantitative immunofluorescence for compartment‐specific and in situ measurement of immune cells in the tumor microenvironment. Furthermore, this quantitative method has been shown to possess better objectivity and reproducibility than conventional semiquantitative analysis,^17^, ^18^ which can also provide more sensitive and superior prognostic information.^17^ However, no studies thus far have attempted to objectively quantify TILs using this method in chordoma.

TIL distribution has been shown to be heterogeneous even among the same tumor tissues.^19^, ^20^, ^21^ Moreover, accumulating evidence indicates that TILs evaluated in different intratumoral regions have distinct roles in the prediction of response to treatment and prognosis.^22^, ^23^, ^24^ Collectively, these data suggest that a separate analysis of TILs in different subareas of the tumors is necessary to obtain a complete and comprehensive understanding of the immune microenvironment in cancer progression. Currently, the immune microenvironment features of chordoma have not been fully elucidated. Prior data have demonstrated lymphocytic infiltration and tumor programmed cell death‐1 ligand 1 (PD‐L1) expression in chordoma tissues.^25^, ^26^, ^27^, ^28^ Moreover, lymphocytic infiltrates and tumoral PD‐L1 expression are correlated with patient outcomes and tumor progression (such as relapse and metastasis) in chordoma.^26^, ^28^ Despite these findings, data on the intratumoral heterogeneity of immune features in the chordoma microenvironment are still lacking. In the present study, we aimed to assess TILs (including programmed death‐1 (PD‐1)^+^, CD3^+^, CD8^+^, CD20^+^, and Foxp3^+^ lymphocytes, where CD is cluster of differentiation) from the tumoral and stromal subregions of chordoma tissues using multiplexed quantitative immunofluorescence and attempted to establish an immune risk score (IRS) model for outcome prediction. We chose to analyze these five immune parameters (described above) because they represented the main immune cell types (T cells, cytotoxic T cells, B cells, and regulatory T lymphocytes) and played a key role in tumor immunity. PD‐1‐positive lymphocytes were analyzed based on the importance of the PD‐1/PD‐L1 pathways in mediating tumor immune evasion.^29^ We analyzed the mutual relationships between TIL data and compared the prognostic performance of the IRS signature with the traditional Enneking staging system as well as other clinicopathologic variables. Finally, we developed a nomogram based on the IRS and clinical prognostic factors for predicting tumor recurrence and death of patients.

## 2 METHODS AND MATERIALS

### 2.1 Patients and tissue samples

In this study, 54 patients with spinal chordoma who received curative resection at our department between June 2002 and April 2015 were recruited as the training cohort for the development of the IRS model. These cohort data have been previously recorded in our studies^27^, ^28^ (Table S1). The sample size in this cohort was calculated as per a previously described method, suggesting at least 10‐15 subjects per predictor variable to produce reasonably stable estimates.^30^ In this study, our final model included four features, and the minimum training data size was 40. For the validation cohort, 23 patients from our institute (between November 2015 and December 2018) and 37 patients from Xiangya Hospital (between February 2006 and October 2018) were included (Table S1). Patients were selected to have tumor characteristics similar to those in the training cohort to ensure comparability (Table S1). For sample size estimation in this group, a power calculation was performed,^31^ and we found that a minimum of 42 patients were required to show differences with a power of 80%, *β* error of 0.2, and *α* error of 0.05. Our validation cohort had 60 patients in total, which was adequate for analysis. The clinical data were retrieved from the patients’ medical records and detailed as we previously described.^27^, ^28^ Patients who had received any types of previous treatments (such as chemotherapy or radiotherapy) and those who had any comorbidities (such as immunocompromised status) on admission were excluded from this study.^28^ Notably, patients with recurrent tumors were also included in both cohorts because (a) previous studies have shown that there are no significant differences for the TIL profiles between the primary and recurrent chordoma tissues^27^, ^28^; (b) chordoma tends to show relapse after surgery, and recurrent patients represent a major population of chordoma patients. Therefore, including recurrent patients in our cohorts may be more representative of the chordoma population in the real world; and (c) our sample size is relatively small in both cohorts. Excluding recurrent patients from our study may further compromise the statistical power and increase the possibility of type II statistical error.

Fresh tumor tissue was collected from five spinal chordoma patients who were treated by surgical resection at our institute in 2019. For subsequent assays, each specimen was separated into two approximately equal halves upon collection. One half was paraffin embedded after formalin fixing for 2 days. The other half was placed in RPMI media on ice for single‐cell suspension preparation.

Formalin‐fixed paraffin‐embedded (FFPE) tissue blocks from 114 patients (for recurrent patients, samples from the first recurrence were used) were retrieved from the Department of Pathology and processed into 4‐μm thick tissue sections. Tumor diagnosis was based on the histological findings in the H&E‐stained tissue sections as per the criteria previously described.^1^ The study was approved by the Institutional Review Board at our hospital, and all patients gave informed consents.

### 2.2 Follow‐up

Patients in the training cohort were followed up by radiographical and clinical examinations until September 2015, while follow‐up information of the validation cohort was updated in April 2019. Tumor recurrence was diagnosed by clinical and imaging findings or histology analysis of specimens from the second surgery.^32^ The primary outcome parameters of interest included local relapse‐free survival (LRFS), measured as the duration from the date of tumor resection to the first local recurrence, and overall survival (OS), which is defined as the time interval from surgery to death from any cause. Observations were censored when the patient was tumor free (LRFS analysis) or alive (OS analysis).

### 2.3 Evaluation of PD‐L1 and Ki‐67 expression as well as the Immunoscore in chordoma tissues

In the training cohort, the Immunoscore pattern (I0‐I4), as well as the expression level of PD‐L1 and Ki‐67 in the chordoma specimens, was obtained from our published data.^27^ In the validation cohort, immunohistochemistry was performed to obtain the published data (Figure S1), in which the same staining procedure, antibodies, and evaluation criteria were used as those for the training cohort to allow for comparability.^27^, ^28^ Given the small sample size, the Immunoscore data were further split into high (I3‐4) and low (I0‐1‐2) groups for analysis according to a previously described method.^33^

### 2.4 Multiplex immunofluorescence

Multiplex immunofluorescence staining was performed as previously reported,^34^, ^35^, ^36^, ^37^ using the Opal 7‐color Manual IHC Kit (PerkinElmer, MA). Briefly, each individual tumor tissue was simultaneously stained with isotype‐specific primary antibodies to detect the tumor mask (cytokeratin 19, PT0087, 1:5000; Immunoway) and microenvironmental TILs, including T lymphocytes (CD3, SP7, 1:10; MXB Biotechnologies Co., Fuzhou, China), regulatory T cells (Foxp3, D2W8E, 1:500; Cell Signaling Technology, Danvers, MA), cytotoxic T cells (CD8, D8A8Y, 1:500; Cell Signaling Technology), B lymphocytes (CD20, PT0029, 1:5000; Immunoway), and PD‐1‐positive lymphocytes (PD‐1, ABT‐PD1, 1:10000; Immunoway). Nuclei were stained with 4′,6‐diamidino‐2‐phenylindole (DAPI; PerkinElmer). Cytokeratin 19 was applied to define the tumor mask as previously suggested.^35^, ^38^

Specifically, the FFPE whole tissue sections were deparaffinized and subjected to antigen retrieval in a pressure cooker containing Tris‐ethylenediaminetetraacetic acid buffer with pH 9.0 for 10 minutes. After antigen blocking in 3% H_2_O_2_ for 15 minutes and in 10% goat serum for 30 minutes at room temperature, the tissue sections were treated with the primary antibodies at 4°C overnight. Then, horseradish peroxidase (HRP)‐conjugated secondary antibodies were incubated at room temperature for 1 hour followed by tyramide‐based HRP activation at 37°C for 20 minutes. Residual HRP activation was quenched by a solution containing 1 mM benzoic hydrazide with 0.15% H_2_O_2_ as previously suggested.^39^ Goat anti‐mouse HRP and Opal 540, Opal 570, or Opal 620 conjugate were used to reveal cytokeratin 19, PD‐1, and CD20, respectively. Similarly, a goat anti‐rabbit HRP and Opal 520, Opal 650, or Opal 690 conjugate was used to detect CD8, Foxp3, and CD3, respectively. Finally, the slides were sealed with coverslips using ProLongGold Antifade reagent with DAPI and allowed to dry overnight.

### 2.5 Automated image analysis

Images were analyzed by inForm software (version 2.1.1, PerkinElmer) as previously documented in the literature.^34^, ^35^ Briefly, the whole tumor sections were scanned by a Vectra system (version 2.0.8, PerkinElmer) with a 4× objective under the same bit depth, laser power, and exposure time to ensure comparability. Then, 10 representative areas of interest (AOIs) were picked from each image of the tumor sample. DAPI identified cell nuclei. The region with cytokeratin 19 positivity was defined as the tumor mask. The stroma subregion excluded the tumor mask from the DAPI area. Quantification of tumoral TILs or stromal TILs was calculated by dividing the density of the positive TILs in the compartment by the area of the corresponding mask, and the data are expressed as positive cells per million pixels.^40^ TIL was recorded as positive when its optical density was above the signal detection threshold (specifically 0.125993 for CD8, 3 for PD‐1, 0.725993 for CD20, 0.289979 for Foxp3, and 0.205993 for CD3), which was defined by the negative controls and visual inspection.^41^ Subsequently, the average of all AOIs for each sample was used for final analysis. Images having less than 3% tumor tissues or with staining artifacts were excluded from the analysis.

### 2.6 Construction of the IRS classifier

Given that a single immune parameter is not optimal for prognosis, and there was significant collinearity among the TIL data (Figure S2A,B), least absolute shrinkage and selection operator (LASSO) Cox regression was exploited to select the most important prognostic immune features for the IRS classifier construction using the training data.^42^ This method was adopted because of its widespread use in variable selection for high‐dimensional data^22^, ^43^ and its ability to overcome overfitting in analyses with collinear data. Specifically, the LASSO analysis uses an L1 penalty to shrink some regression coefficients toward zero. The larger the penalty value λ (also called the tuning parameter), the fewer the number of prognostic factors selected. We used 10‐time cross‐validations to determine the optimal value of λ via minimum criteria in which the value of λ was related to the lowest partial likelihood deviance.

Subsequently, the IRS was developed for each patient by integrating the quantitative density for each feature chosen by the LASSO analysis with the corresponding regression coefficient from the penalized LASSO Cox regression model. We investigated the predictive accuracy of the IRS and compared the prognostic performance of this classifier with each immune feature, the Immunoscore, the Enneking staging system, and other classic clinical variables.

### 2.7 Flow cytometry

Flow cytometry was conducted as previously described.^18^ Briefly, single‐cell suspensions were dissociated from fresh chordoma tissues and passed through a 70‐mm nylon cell strainer (Corning). The cell suspension was centrifuged, and the pellet was resuspended to 10^7^ cells/mL in phosphate‐buffered saline containing 5% fetal bovine serum and 0.1% sodium azide. Then, the cells were split into five equal batches of 10^6^ cells and incubated on ice for 20 minutes with the Fc Receptor Binding Inhibitor (eBioscience). One batch was treated with a mix of anti‐CD3 allophycocyanin (APC)‐CY7‐conjugated (0.1 mg/mL; BD Biosciences), Percp‐conjugated anti‐CD8 (0.05 mg/mL; BD Biosciences), anti‐CD20 fluorescein isothiocyanate conjugated (0.012 mg/mL; eBioscience), anti‐PD‐1 APC‐conjugated (0.1 mg/mL; BD Biosciences), and anti‐Foxp3 phycoerythrin‐conjugated antibodies (0.1 mg/mL; eBioscience), followed by fix and permeabilization buffer (eBioscience) for 30 minutes in the dark on ice. The other four batches were used as compensation controls, one unstained and three stained in parallel with the antibody mix. After washing, flow cytometric analysis of CD3‐, CD8‐, Foxp3‐, CD20‐, and PD‐1‐positive subpopulations was performed on a FACSCanto II Flow Cytometer (BD Biosciences), and the data were collected by FACSDiva software. Finally, flow cytometry plots were produced and analyzed using FlowJo software (Tree Star, Ashland, OR).

### 2.8 Statistical analysis

Quantitative (expressed as the mean ± standard deviation) and categorical data were analyzed by one‐way analysis of variance or Student's *t*‐test and Wilcoxon's rank sum test or chi‐square test, respectively. X‐tile software (version 3.6.1) was used to obtain the cutoff points for variables in the survival analysis with OS as the outcome parameter,^22^, ^44^ where the *P* value from the log‐rank test was corrected accordingly.^44^ The Kaplan‐Meier curve was used to display the LRFS and OS curves, and differences in survival probabilities between subgroups were compared by the log‐rank test. The Cox proportional hazard model was performed for multivariate survival analysis. Bootstrap analysis was used to test the robustness of the IRS as a prognostic predictor.^45^, ^46^ With combined data from the training and validation cohorts, the nomogram was constructed based on the IRS and previously reported significant clinical factors,^2^, ^47^, ^48^ as well as the results from our multivariate analysis. The nomogram's predictive accuracy was determined by a concordance index (C‐index) and receiver operating characteristic (ROC) curve, which was also applied to assess the sensitivity and specificity of the variables for LRFS and OS prediction. A calibration curve was plotted for the actual observed survival versus predicted probabilities from the nomogram. Decision curve analysis was performed to explore the clinical usefulness of the nomogram model. Statistical analyses were performed using R version 3.5.1 (R Foundation for Statistical Computing, Vienna, Austria). All tests were two‐sided, and a *P* value ≤ .05 was considered significant.

## 3 RESULTS

### 3.1 Patient clinicopathological characteristics

A total of 114 patients (54 in the training cohort and 60 in the validation cohort) were included. The patient characteristics are detailed in Table S1. In brief, the training cohort had 19 females and 35 males. Among them, 11 patients had recurrent chordomas, and 43 patients had primary diseases. Enneking appropriate (EA) and Enneking inappropriate (EI) tumor resections were performed in 36 and 18 patients, respectively. In the validation cohort, there were 42 males and 18 females. Forty‐seven had primary tumors, and 13 had tumors at relapse. Of them, 36 patients underwent EA resection, and 24 received EI resection. Histologically, all chordoma cases were of the classic type.

### 3.2 Objective quantitative analysis of the TILs in the chordoma tissues

**FIGURE 1:**
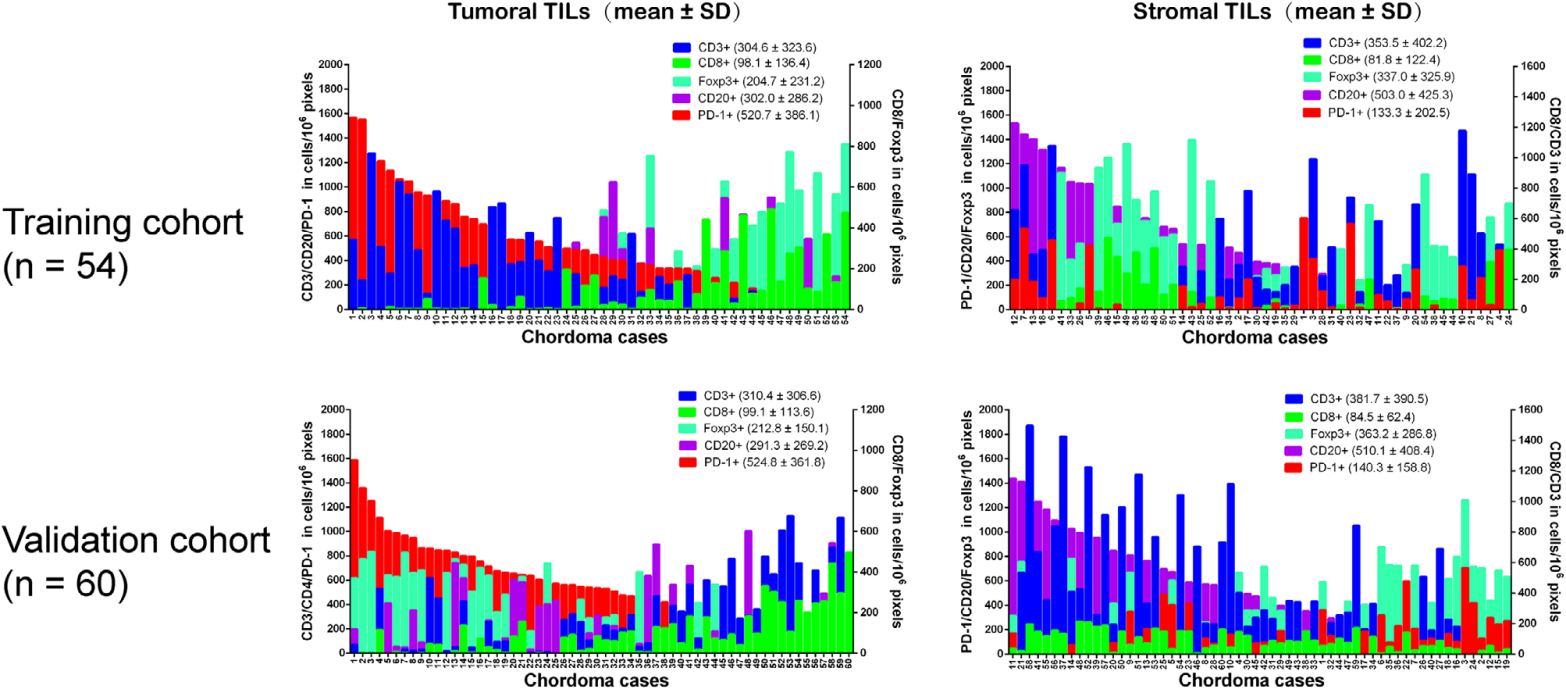
Quantitative levels of tumor‐infiltrating lymphocytes subtypes in tumoral and stromal subareas of chordoma specimens in the training and validation cohort.

**FIGURE 2:**
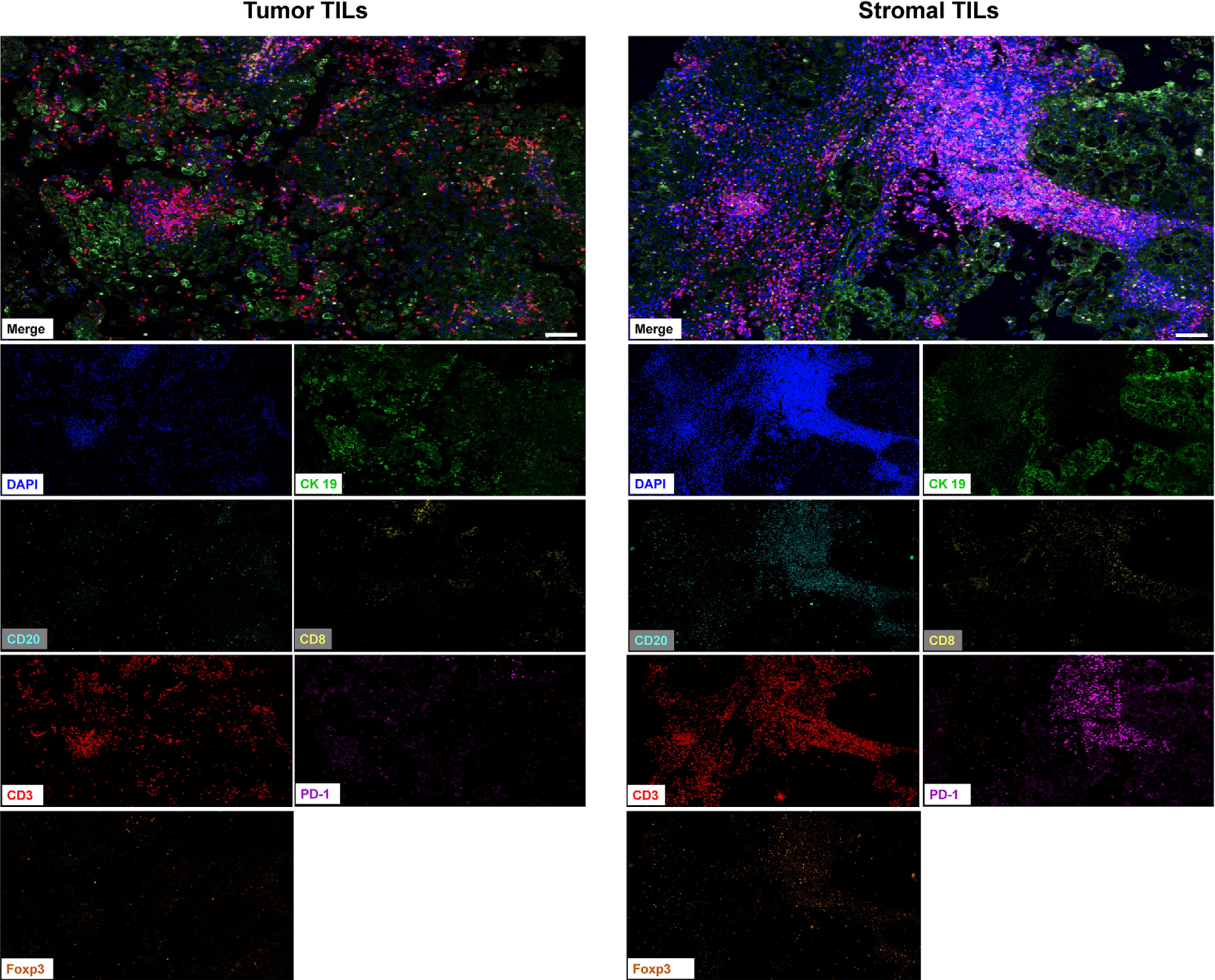
Representative immunofluorescence images showing tumor‐infiltrating lymphocytes level within tumor and stromal subregions of chordoma tissues. Scale bar = 50 μm. For multiplex immunofluorescence staining, 4‐μm thick tumor sections were dewaxed and rehydrated. After antigen retrieval and blocking, tissue sections were treated with primary cytokeratin 19 antibody (1:5000). HRP‐conjugated secondary antibodies were then incubated at room temperature for 1 hour followed by tyramide‐based HRP activation at 37°C for 20 min. This was repeated five more times using the following antibodies: CD3 (1:10), Foxp3 (1:500), CD8 (1:500), CD20 (1:5000), and PD‐1 (1:10000). Nuclei were stained with DAPI.

Quantitative densities and representative images of TIL subtypes in the chordoma tissues are shown in Figures 1 and 2, respectively. The TIL data showed good concordance between the training and validation cohorts. Moreover, there were significant differences in the TIL distribution in both the tumoral and stromal subareas (training cohort: both *P* < .001; validation cohort: both *P* < .001). In the tumor subregion, the PD‐1^+^ TILs showed the highest extent of infiltration, whereas the CD8^+^ TILs had the lowest level. In the stromal compartment, however, the CD20^+^ TILs displayed the highest infiltration density, whereas the CD8^+^ TILs still showed the lowest number. Notably, intratumoral heterogeneity was also seen for the infiltration pattern of the same TIL subset (Table S2). Specifically, the CD8^+^ and PD‐1^+^ TILs were higher in the tumoral region than in the stromal region. In contrast, the densities of the stromal CD3^+^, CD20^+^, and Foxp3^+^ TILs were higher than their counterparts within the tumoral compartment.

### 3.3 Validation of quantitative immunofluorescence assay parameters

**FIGURE 3:**
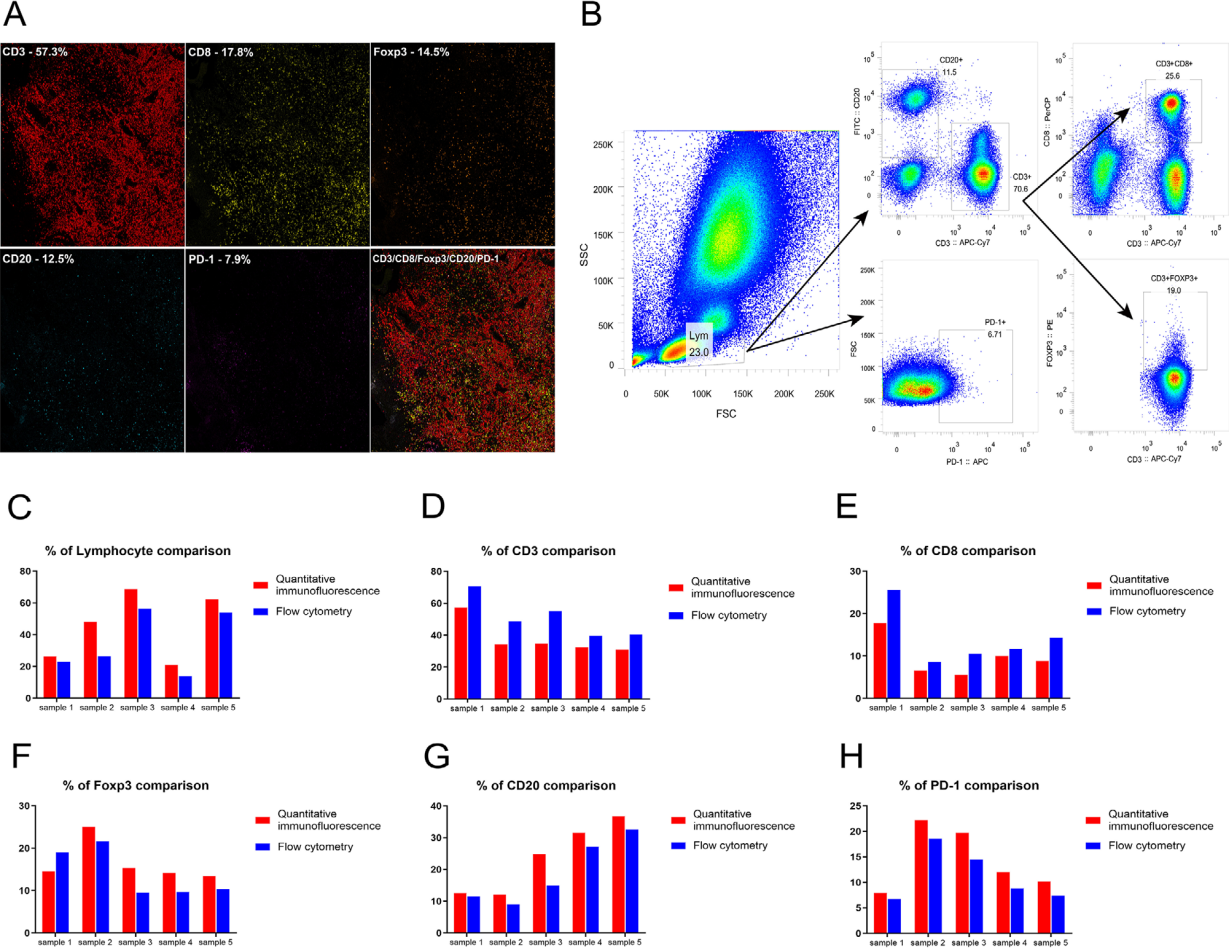
Validation of quantitative immunofluorescence assay for measuring TILs levels by comparison to flow cytometry on fresh chordoma samples. A, Representative field of view showing TILs subpopulation proportions from quantitative immunofluorescence on chordoma tissue. B, Representative example of flow cytometry on chordoma tissue, in which numbers in this picture represent the percentage of TILs analyzed. The total lymphocytes (left) were gated into PD‐1 subpopulation, distinct CD3 and CD20 populations (middle), and overlapping CD3 and CD8, as well as CD3 and Foxp3 populations (right). C‐H, Percentage of lymphocytes and subpopulations was similar when analyzed by flow cytometry and quantitative immunofluorescence. For multiplex immunofluorescence staining, 4‐μm‐thick tumor sections were dewaxed and rehydrated. After antigen retrieval and blocking, tissue sections were treated with primary cytokeratin 19 antibody (1:5000). HRP‐conjugated secondary antibodies were then incubated at room temperature for 1 hour followed by tyramide‐based HRP activation at 37°C for 20 min. This was repeated five more times using the following antibodies: CD3 (1:10), Foxp3 (1:500), CD8 (1:500), CD20 (1:5000), and PD‐1 (1:10000). Nuclei were stained with DAPI. For flow cytometry, single‐cell suspensions with a concentration of 10^7^ cells/mL in phosphate‐buffered saline [PBS] (containing 5% fetal bovine serum [FBS] and 0.1% sodium azide) were split into five equal proportions of 10^6^ cells. One proportion was treated with a mix of anti‐CD3 APC‐CY7‐conjugated (0.1 mg/mL), anti‐CD8 Percp‐conjugated (0.05 mg/mL), anti‐CD20 fluorescein isothiocyanate conjugated antibodies (0.012 mg/mL), anti‐PD‐1 APC‐conjugated antibodies (0.1 mg/mL), anti‐Foxp3 PE‐conjugated antibodies(0.1mg/mL), and fix and permeabilization buffer for 30 min in the dark on ice. The other four proportions were used as compensation controls. Flow cytometry consistently determined a greater proportion of CD3‐ and CD8‐positive lymphocytes, while quantitative immunofluorescence consistently resulted in a greater proportion of lymphocytes, B cells, as well as PD‐1^+^ and Foxp3^+^ T cells.

Five chordoma specimens were used for analysis of PD‐1, CD3, CD8, CD20, and Foxp3 expression by both flow cytometry and quantitative immunofluorescence. The percentage of lymphocytic infiltrates was calculated for each of 10 representative AOIs in each entire tumor specimen (Figure 3A) using a quantitative immunofluorescence assay, and the average was obtained for analysis. Flow cytometry of the same samples demonstrated the presence of lymphocytes in tumor tissues with varying densities (Figure 3B). Further results revealed a good concordance of the lymphocyte percentage by both methods (Figure 3C‐H). Specifically, the lymphocyte percentage ranged from 13.9% to 56.3% by flow cytometry and 21.0% to 68.7% by multiple immunofluorescence. Flow cytometry consistently showed a higher proportion of CD3‐ and CD8‐positive lymphocytes, while quantitative immunofluorescence uniformly reported a greater proportion of lymphocytes, B cells, as well as PD‐1+ and Foxp3+ T cells. In all cases, the CD3‐positive lymphocytes were relatively abundant, and the CD8‐ or Foxp3‐positive T cells were a small proportion of the CD3^+^ cells.

### 3.4 Construction and description of the IRS prognostic model

With the optimal λ value of 1.778 × 10^–3^ in the LASSO analysis (Figure S2C), four immune features (sCD8^+^, sFoxp3^+^, tFoxp3^+^, and tPD‐1^+^ TILs) were selected for the IRS construction (Figure S2D). The coefficients for the above four immune parameters in LASSO Cox regression were 7.27 × 10^–4^, 9.126 × 10^–4^, −4.463 × 10^–3^, and 3.471 × 10^–3^, respectively. Therefore, the IRS was calculated for each patient as follows: IRS = density of sCD8^+^ TILs × 7.27 × 10^–4^ + density of sFoxp3^+^ TILs × 9.126 × 10^–4^ + density of tFoxp3^+^ TILs × (−4.463 × 10^–3^) + density of tPD‐1^+^ TILs × 3.471 × 10^–3^. The mean values for the IRS in the training and validation cohorts were 1.26 ± 1.96 and 1.27 ± 1.01, respectively.

### 3.5 Association between IRS and patient outcome

**FIGURE 4:**
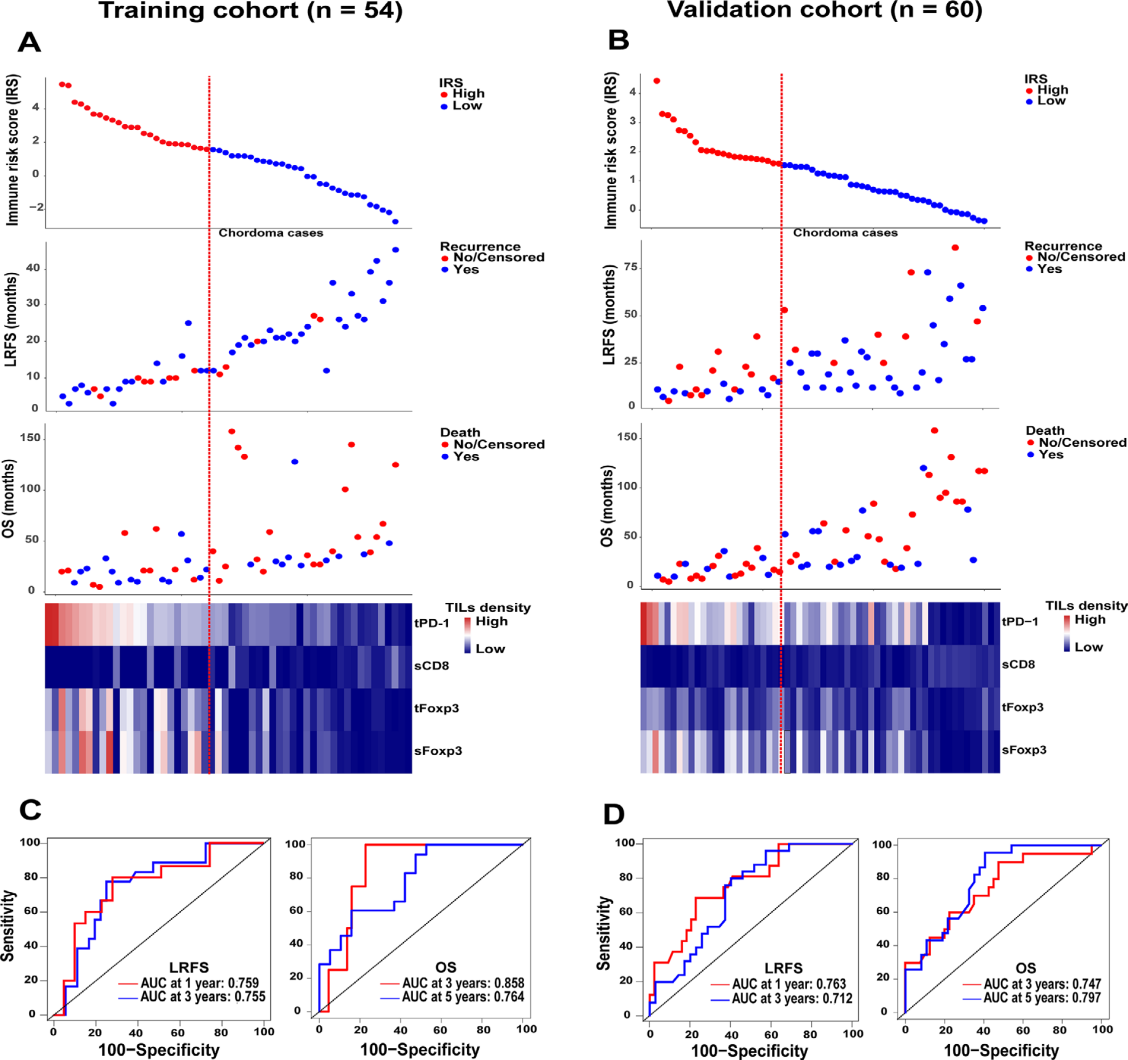
Distribution of IRS, recurrence status, and survival status among the training (A) and validation (B) chordoma samples. The heatmap (bottom) shows the profile of four immune features included for the IRS construction in chordoma patients. Columns represent patients who were sorted descendingly by their IRS levels. Red dotted line represents the IRS cutoff dividing patients into high and low subgroups. B, Receiver operating characteristics curves show the sensitivity and specificity for the IRS (as a continuous variable) in predicting LRFS and OS of patients from the training (C) and validation (D) cohort. Abbreviations: AUC, area under the curve; IRS, immune risk score; LRFS, local recurrence‐free survival; OS, overall survival; sCD8, stromal CD8^+^ TILs; sFoxp3, stromal Foxp3^+^ TILs; tFoxp3, tumor Foxp3^+^ TILs; TILs, tumor‐infiltrating lymphocytes; tPD‐1, tumor PD‐1^+^ TILs.

The distribution of the recurrence and survival status as well as the IRS of the patients is illustrated in Figure 4A,B. ROC analysis showed that the IRS as a continuous variable performed well in predicting LRFS and OS in both cohorts (Figure 4C,D). Using X‐tile software, we found that the prognostic cutoff point for IRS in relation to OS was 1.57 (Figure S3). The patients were then separated into low (≤1.57) and high IRS (>1.57) groups according to this threshold value.

**FIGURE 5:**
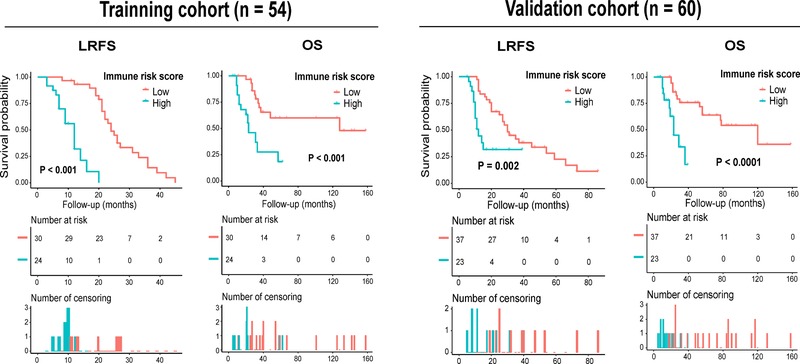
Kaplan‐Meier curves of LRFS and OS of spinal chordoma patients from the training and validation cohort stratified by the IRS. Abbreviations: IRS, immune risk score; LRFS, local recurrence‐free survival; OS, overall survival.

Univariate Kaplan‐Meier analysis disclosed that high IRS patients harbored worse LRFS than those with a low IRS (Figure 5). Similarly, in the analysis of OS, patients with a low IRS had a significantly better survival than the patients with a high IRS (Figure 5). In addition, the four immune parameters showed significant associations with both patients’ LRFS and OS after cutoff determination using X‐tile software (Figures S3‐S5). Multivariate Cox regression analysis with adjustment for clinical predictors found that the IRS independently predicted both LRFS and OS of patients (Figure S6A‐D). Further bootstrap analysis confirmed the reliability of IRS as a prognostic factor in both univariate and multivariate analyses (Figure S6E‐H).

### 3.6 Association between the IRS classifier and clinicopathologic variables

In both cohorts, the IRS was found to have a positive association with tumor invasion into the surrounding muscle tissues, PD‐L1 expression on tumor cells, and type of surgical resection (Figure S7 and Tables S3 and S4). Similarly, in the training cohort, a significant or borderline significant association was also detected between the IRS and overall TIL level and between the IRS and patient age (Figure S7 and Table S3). However, tumors with a high IRS were more likely to have advanced Enneking staging than those with a low IRS in the validation cohort, although this correlation was not significant (Figure S7 and Table S4).

Subgroup analysis was also performed for the IRS and four clinicopathologic variables (age, type of surgery, tumor muscle invasion, and Enneking staging), which were all previously reported to influence the LRFS and OS of patients with spinal chordoma. Our results revealed that the IRS still displayed good prognostic performance in predicting LRFS and/or OS of the patients stratified by the four clinical factors (Figures S8‐S11).

### 3.7 Comparison of the IRS classifier with four immune features, the Immunoscore, Enneking staging system, and other clinicopathologic parameters in predicting survival

In both cohorts, ROC analysis found that the IRS classifier could accurately reflect prognosis and also had stronger predictive power than the four immune features included for IRS construction (Figure S12A‐D). Additionally, our analysis showed that the IRS had a greater prognostic accuracy than the Immunoscore, Enneking staging system and other clinical factors in predicting LRFS and OS (Figure S12E‐H). Importantly, the combined IRS and Enneking staging model showed an improved ability compared to using each model alone for outcome prediction (Figure S12E‐H).

### 3.8 Establishment of the LRFS and OS prediction nomogram

**FIGURE 6:**
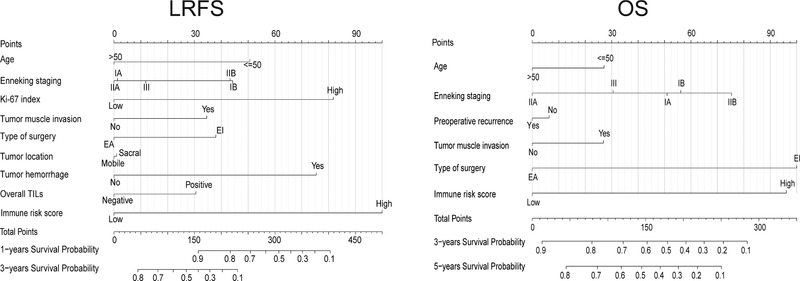
Establishment of the nomogram based on the IRS and clinicopathological parameters for LRFS and OS prediction using the combined data from training and validation cohort (n = 114). The clinical factors were selected based on literature reports and the results from our multivariable Cox analysis, depending on their significant association with LRFS or OS of patients, respectively. Abbreviations: IRS, immune risk score; LRFS, local recurrence‐free survival; OS, overall survival.

**FIGURE 7:**
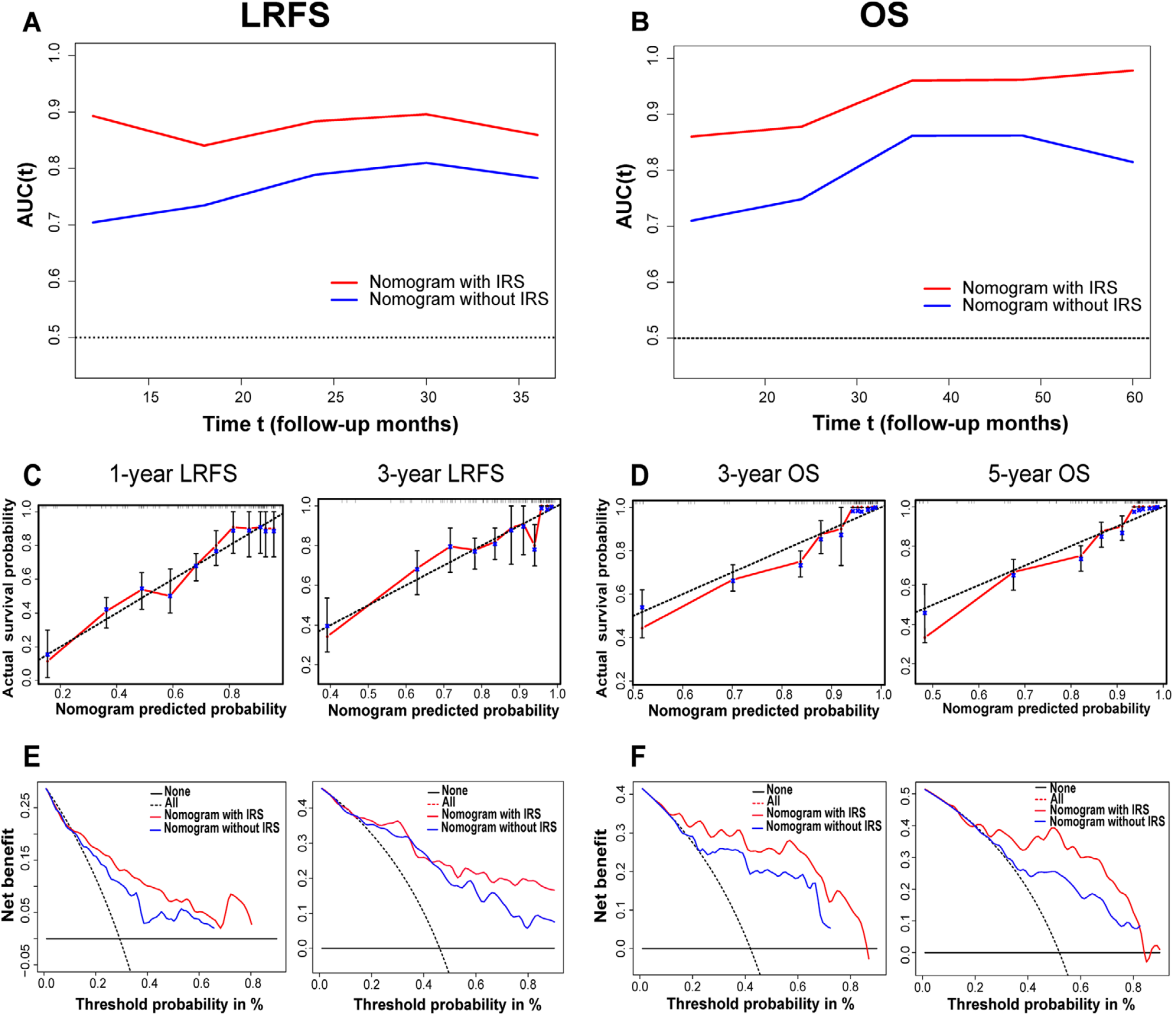
Time‐dependent receiver operating characteristic curves show sensitivity and specificity for the nomogram model in predicting LRFS (A) and OS (B) of patients. The calibration curves depict the agreement between predicted and observed LRFS (C) and OS (D) probability. Decision curve analysis of the nomogram for LRFS (E) and OS (F). Abbreviations: LRFS, local recurrence‐free survival; OS, overall survival.

To provide a clinically relevant quantitative tool for survival prediction, we developed the LRFS and OS nomogram including the IRS and significant clinical factors associated with patient survival (Figure 6). The IRS‐based nomogram exhibited high predictive accuracy with a C‐index of 0.811 (95% CI: 0.756‐0.867) and 0.793 (95% CI: 0.737‐0.849) for 1‐year LRFS and 3‐year LRFS, respectively. Similar outcomes were also observed in terms of 3‐year OS (C‐index: 0.830, 95% CI: 0.784‐0.877) and 5‐year OS (C‐index: 0.790, 95% CI: 0.738‐0.842). Subsequent time‐dependent ROC analysis further validated these results (Figure 7A,B). In addition, calibration plots showed that the nomogram‐predicted survival probabilities fit well with the actual observations (Figure 7C,D). Moreover, decision curve analysis demonstrated a high clinical net benefit for the IRS‐based nomogram across a broad range of threshold probabilities (Figure 7E,F).

## 4 DISCUSSION

Preceding reports on TIL characterization and their associations with prognosis have been documented in the literature for chordoma.^27^, ^28^, ^49^ However, these studies usually use H&E‐based pathologist estimation or single‐color immunohistochemical technology for TIL determination, which is subjective with variable reproducibility. Furthermore, the traditional strategy lacks compartment specificity and is unable to simultaneously characterize multiple cell subtypes. In this study, we used multiplex quantitative immunofluorescence to enable objective and compartment‐specific measurement of the immune cells in the chordoma microenvironment and developed an immune feature–based risk score model for outcome prediction. We reported that the IRS composed of tumoral PD‐1^+^ and Foxp3^+^ TILs as well as stromal CD8^+^ and Foxp3^+^ TILs was significantly correlated with the survival of spinal chordoma patients. More importantly, a nomogram integrating the IRS and important clinical parameters accurately reflected prognosis and also showed better predictive performance than each nomogram alone. These data highlight the central role of the local adaptive immune response in determining prognosis^33^ and provide further support for the use of immunotherapy to treat chordoma.^27^, ^28^, ^49^, ^50^

Previous studies have indicated that tumor PD‐L1 expression and preexisting microenvironmental TIL levels are effective biomarkers in forecasting the response to immune checkpoint blockade.^13^, ^14^, ^51^ Our data showed that the IRS was associated with tumoral PD‐L1 expression in both cohorts and related to overall TIL densities in the training sets, which suggests the potential use of the IRS to predict patient response to immunotherapy in spinal chordoma. Further investigations addressing the IRS data before and after immunotherapy of these patients are required to test this idea. Interestingly, we also observed that the number of intratumoral immune infiltrates was nonhomogeneous across the dimensions of a single section, which was consistent with previous reports.^19^, ^52^ This immune heterogeneity deserves further elucidation and may also indicate the importance of obtaining not only cell abundance but also spatial data in the assessment of microenvironmental TILs.^52^ In addition, we found that the TILs distributed in the tumoral or stromal subregions were closely correlated in chordoma tissues, particularly for the same TIL subsets. This finding may suggest a similar cellular origin or spatiotemporally dynamic migration for intratumoral and stromal TILs during tumor progression. Our future work will use migration and/or invasion tests to evaluate the effect of TIL displacement between the intratumoral and stromal subregions on chordoma progression.

Accurate prognostic assessment is important for selecting appropriate therapy and customizing postoperative monitoring in cancer patients. Although the Enneking staging system is routinely used in clinical practice to notify risk stratification for spinal chordoma, clinical outcomes vary even among patients with the same staging, suggesting that this system alone is not adequate for prognosis. In this study, we developed a risk classifier comprising four immune features and found it to be a powerful and independent prognostic tool that also had a superior predictive performance than the widely used Enneking system. These findings are consistent with previous observations^22^, ^53^, ^54^ and support our earlier findings that measurement of microenvironmental TILs is a reliable and clinically applicable procedure to predict chordoma outcome.^27^, ^28^, ^49^ Although the Immunoscore has been recently shown to yield a more powerful prediction than the Enneking staging in chordoma,^28^ our study showed that the IRS model had significant advantages over the Immunoscore system in predicting patient outcome. This phenomenon may be explained by the fact that our immune feature–based classifier had a more comprehensive immunologic evaluation and was established using a LASSO Cox regression method, which likely improved its predictive accuracy. In addition, our analysis found that the combined IRS and Enneking system had better prognostic value compared to their individual use. These outcomes indicate that the immune features can add additional value to the Enneking stage, thus reinforcing its prognostic ability. In addition, as the immune classifier reflects the bioimmunological characteristics of tumors and provides different information from the Enneking system, subsequent inclusion of this classifier into Enneking staging may represent a new strategy for the classification of chordoma, called the Enneking‐Immune system, similar to previous publications.^22^, ^55^, ^56^, ^57^ Notably, our study displayed a significant survival difference between patients with similar clinicopathologic features who were stratified by the IRS classifier. These findings suggest that the identification of tumor‐infiltrated immune cells may enable clinicians to obtain a more exhaustive and precise knowledge of chordoma prognosis, thereby optimizing treatment strategies and improving survival.

Currently, there is no effective tool for outcome prediction in chordoma.^28^ In the present study, we established a nomogram to predict survival based on the IRS and clinical factors. We disclosed that the nomogram had good performance in predicting prognosis and was more accurate than the IRS signature and clinicopathologic parameters, including the traditional Enneking classification. Moreover, the prognostic accuracy of our nomogram was confirmed in the validation cohort. These results may allow oncologists to accurately predict chordoma outcome and can also be useful in identifying patients with high‐risk disease who are candidates for aggressive therapy.

### 4.1 Limitations

More prospective studies involving large number of patients are needed to further confirm the role of the IRS model in spinal chordoma prognosis. Currently, we have established a spinal chordoma database using combined data sets and are also attempting to prospectively add data for each newly diagnosed case in this database. After including enough newly diagnosed patients, we can further test the reliability of the IRS model in chordoma prognosis. In addition, with this database, we can assess the ability of the IRS model in drug response prediction, therapy monitoring and evaluation of chordoma patients after prospectively obtaining IRS data before and after treatment. Moreover, our study did not characterize circulating immune cells in chordoma patients, although these features may be relevant to prognosis and the microenvironmental lymphocytic profile. In addition, we should note that our study lacked experimental data to elucidate the exact mechanisms of how immune cells affect the clinical outcomes of patients. Finally, several other immune features, such as CD45RA, CD57, CD66b, CD68, and IL17, may also be evaluated for their expression profile in chordoma tissues, as well as for eligibility for inclusion in the immune classifier and nomogram, as they have been shown to be indicative of cancer prognosis.^22^ The current study did not analyze these markers due to limited fluorescence channels available.

## 5 CONCLUSIONS

The present study demonstrated that the IRS model consisting of four immune variables was associated with clinicopathologic features and could effectively predict recurrence and survival of patients with spinal chordoma. The IRS classifier provided superior prognostic value to traditional clinical predictors and was an essential complement for Enneking staging. Moreover, the IRS‐based nomogram showed adequate performance in predicting survival. These findings suggest that the IRS may prove to be a useful tool for prognostic risk stratification in spinal chordoma and facilitate personalized therapy decision making for patients.

## ETHICAL APPROVAL

The study protocol was approved by the Institutional Review Board at the Second Xiangya Hospital of Central South University, Hunan, China. Written informed consent was obtained from each patient for publication of this study.

## CONFLICT OF INTEREST

The authors declare that there is no conflict of interest that could be perceived as prejudicing the impartiality of the research reported.

## AUTHOR CONTRIBUTIONS

Study concept and design: Zou, Wang, Lv, and Li. Analysis and interpretation of data: Zou, Pan, Huang, Zhang, Jiang, and She. Zou, Pan, and Wang performed the experiments. Drafting of the manuscript: Zou, Zhang, Escobar, and Li. All authors participated in data acquisition and read and approved the final manuscript.

## Supporting information

Supplementary Information

## Data Availability

All data relevant to the study are included in the article or uploaded as Supporting Information.
