# Supplementary material for: A four‐factor immune risk score signature predicts the clinical outcome of patients with spinal chordoma
Source: Clin Transl Med. 2020 May 13;10(1):224–37. doi: 10.1002/ctm2.4 (PMC7240847; doi:10.1002/ctm2.4)
Supplement: Supplementary file 1 — Supplementary Information [file CTM2-10-224-s001.doc]

**Supplementary Table 1** Comparison of the baseline characteristics between the training and validation cohort

| Characteristic | Training cohort (n = 54) | Validation cohort (n = 60) | Test statistic | *P-*value |
| --- | --- | --- | --- | --- |
| Age (years) |  |  |  |  |
| ≤ 50 | 23 (42.6%) | 20 (33.3%) | 1.037 | 0.308 |
| > 50 | 31 (57.4%) | 40 (66.7%) |  |  |
| Sex |  |  |  |  |
| Male | 35 (64.8%) | 42 (70%) | 0.349 | 0.555 |
| Female | 19 (35.2%) | 18 (30%) |  |  |
| Tumor size |  |  |  |  |
| ≤ 5 cm | 21 (38.9%) | 22 (36.7%) | 0.060 | 0.807 |
| > 5 cm | 33 (61.1%) | 38 (63.3%) |  |  |
| Tumor location |  |  |  |  |
| Sacral vertebra | 42 (77.8%) | 45 (75%) | 0.121 | 0.728 |
| Mobile spine | 12 (22.2%) | 15 (25%) |  |  |
| Surrounding  muscle invasion |  |  |  |  |
| Yes | 36 (66.7%) | 31 (51.7%) | 2.639 | 0.104 |
| No | 18 (33.3%) | 29 (48.3%) |  |  |
| Preoperative recurrence |  |  |  |  |
| Yes | 11 (20.4%) | 13 (21.7%) | 0.029 | 0.865 |
| No | 43 (79.6%) | 47 (78.3%) |  |  |
| Grade |  |  |  |  |
| High | 38 (70.4%) | 42 (70%) | 0.002 | 0.966 |
| Low | 16 (29.6%) | 18 (30%) |  |  |
| Enneking staging |  |  |  |  |
| IA + IB + IIA | 23 (42.6%) | 33 (55%) | 1.751 | 0.186 |
| IIB + III | 31 (57.4%) | 27 (45%) |  |  |
| Type of resection |  |  |  |  |
| EI | 18 (33.3%) | 24 (40%) | 0.543 | 0.461 |
| EA | 36 (66.7%) | 36 (60%) |  |  |
| Tumor hemorrhage |  |  |  |  |
| No | 10 (18.5%) | 17 (28.3%) | 1.515 | 0.218 |
| Yes | 44 (81.5%) | 43 (71.7%) |  |  |
| Tumor necrosis |  |  |  |  |
| Absent + Mild | 31 (57.4%) | 33 (55%) | 0.067 | 0.796 |
| Moderate + Severe | 23 (42.6%) | 27 (45%) |  |  |
| Ki-67 index |  |  |  |  |
| Low | 25 (46.3%) | 26 (43.3%) | 0.101 | 0.751 |
| High | 29 (53.7%) | 34 (56.7%) |  |  |
| Tumor PD-L1 |  |  |  |  |
| Positive | 37 (68.5%) | 40 (66.7%) | 0.044 | 0.833 |
| Negative | 17 (31.5%) | 20 (33.3%) |  |  |
| Lobular pattern growth |  |  |  |  |
| Yes | 21 (38.9%) | 25 (41.7%) | 0.091 | 0.763 |
| No | 33 (61.1%) | 35 (58.3%) |  |  |
| Overall TILs |  |  |  |  |
| Positive | 31 (57.4%) | 34 (56.7%) | 0.006 | 0.936 |
| Negative | 23 (42.6%) | 26 (43.3%) |  |  |
| Follow-up duration (months) | 42.4 ± 38.9 | 43.4 ± 36.9 | -0.145 | 0.885 |

EI, Enneking inappropriate; EA, Enneking appropriate; TILs, tumor-infiltrating lymphocytes; PD-L1,

programmed cell death-1 ligand 1.

**Supplementary Table 2** Distribution of TILs subtypes in tumoral and stromal subregions of spinal chordoma tissues.

| TILs subtypes | Training cohort (n = 54) | | Test statistics | *P*-value | Validation cohort (n = 60) | | Test statistics | *P*-value |
| --- | --- | --- | --- | --- | --- | --- | --- | --- |
|  | Tumoral subarea (mean ± SD, cells/10^6^ pixels ) | Stromal subarea (mean ± SD, cells/10^6^ pixels ) |  |  | Tumoral subarea (mean ± SD, cells/10^6^ pixels ) | Stromal subarea (mean ± SD, cells/10^6^ pixels ) |  |  |
| PD-1 | 520.7 ± 386.1 | 133.3 ± 202.5 | t = 10.330 | **< 0.001** | 524.8 ± 361.8 | 140.4 ± 158.9 | t = 7.536 | **< 0.001** |
| CD8 | 98.1 ± 136.4 | 81.9 ± 122.4 | t = 1.044 | 0.301 | 99.1 ± 113.7 | 84.5 ± 62.4 | t = 0.869 | 0.387 |
| CD20 | 302.0 ± 286.1 | 503.1 ± 425.3 | t = -3.595 | **0.001** | 291.3 ± 269.1 | 510.2 ± 408.4 | t = 3.466 | **< 0.001** |
| Foxp3 | 204.6 ± 231.2 | 337.0 ± 325.9 | t = -3.505 | **0.001** | 212.8 ± 150.1 | 363.2 ± 286.8 | t = 3.600 | **< 0.001** |
| CD3 | 304.6 ± 323.6 | 353.5 ± 402.2 | t = -2.474 | **0.017** | 310.4 ± 306.6 | 381.7 ± 390.5 | t = 1.112 | 0.268 |

Bold indicates *P* < 0.05; TILs, tumor-infiltrating lymphocytes; SD, standard deviation; PD-1, programmed cell death-1.

**Supplementary Table 3** Association between Immune risk score and clinicopathological features of spinal chordoma patients in the training cohort (n = 54)^a^

| Clinicopathological factors | No. of patients (n = 54) | Immune risk score | | | Test statistic | *P-*value |
| --- | --- | --- | --- | --- | --- | --- |
|  |  | Low (%) | High (%) | |  |  |
| Age (years) |  |  | |  |  |  |
| ≤ 50 | 23 | 16 (70%) | | 7 (30%) | 3.185 | 0.074 |
| > 50 | 31 | 14 (45.2%) | | 17 (54.8%) |  |  |
| Sex |  |  | |  |  |  |
| Male | 35 | 18 (51.4%) | | 17 (48.6%) | 0.685 | 0.407 |
| Female | 19 | 12 (63.2%) | | 7 (36.8%) |  |  |
| Tumor size |  |  | |  |  |  |
| ≤ 5 cm | 21 | 12 (57.1%) | | 9 (42.9%) | 0.035 | 0.851 |
| > 5 cm | 33 | 18 (54.5%) | | 15 (45.5%) |  |  |
| Tumor location |  |  | |  |  |  |
| Sacral vertebra | 42 | 23 (54.8%) | | 19 (45.2%) | 0.048 | 0.826 |
| Mobile spine | 12 | 7 (58.3%) | | 5 (41.7%) |  |  |
| Surrounding  muscle invasion |  |  | |  |  |  |
| Yes | 36 | 15 (41.7%) | | 21 (58.3%) | 8.438 | **0.004** |
| No | 18 | 15 (83.3%) | | 3 (16.7%) |  |  |
| Preoperative recurrence |  |  | |  |  |  |
| Yes | 11 | 6 (54.5%) | | 5 (45.5%) | 0.000 | 1.000 |
| No | 43 | 24 (55.8%) | | 19 (44.2%) |  |  |
| Grade |  |  | |  |  |  |
| High | 38 | 22 (57.9%) | | 16 (42.1%) | 0.284 | 0.594 |
| Low | 16 | 8 (50%) | | 8 (50%) |  |  |
| Enneking staging |  |  | |  |  |  |
| IA + IB + IIA | 23 | 12 (52.2%) | | 11 (47.8%) | 0.186 | 0.667 |
| IIB + III | 31 | 18 (58.1%) | | 13 (41.9%) |  |  |
| Type of resection |  |  | |  |  |  |
| EI | 18 | 4 (22.2%) | | 14 (77.8%) | 12.150 | **< 0.001** |
| EA | 36 | 26 (72.2%) | | 10 (27.8%) |  |  |
| Tumor hemorrhage |  |  | |  |  |  |
| No | 10 | 6 (60%) | | 4 (40%) | 0.000 | 1.000 |
| Yes | 44 | 24 (54.5%) | | 20 (45.5%) |  |  |
| Tumor necrosis |  |  | |  |  |  |
| Absent + Mild | 31 | 17 (54.8%) | | 14 (45.2%) | 0.015 | 0.902 |
| Moderate + Severe | 23 | 13 (56.5%) | | 10 (43.5%) |  |  |
| Ki-67 index |  |  | |  |  |  |
| Low | 25 | 16 (64%) | | 9 (36%) | 1.344 | 0.246 |
| High | 29 | 14 (48.3%) | | 15 (51.7%) |  |  |
| Tumor PD-L1 |  |  | |  |  |  |
| Positive | 37 | 15 (40.5%) | | 22 (59.5%) | 10.761 | **0.001** |
| Negative | 17 | 15 (88.2%) | | 2 (11.8%) |  |  |
| Lobular pattern growth |  |  | |  |  |  |
| Yes | 21 | 11 (52.4%) | | 10 (47.6%) | 0.140 | 0.708 |
| No | 33 | 19 (57.6%) | | 14 (42.4%) |  |  |
| Overall TILs |  |  | |  |  |  |
| Positive | 31 | 12 (38.7%) | | 19 (61.3%) | 8.365 | **0.004** |
| Negative | 23 | 18 (78.3 %) | | 5 (21.7%) |  |  |

Bold indicates *P* < 0.05.

EI, Enneking inappropriate; EA, Enneking appropriate; TILs, tumor-infiltrating lymphocytes; PD-L1,

programmed cell death-1 ligand 1; ^a^analyzed by Chi-square test.

**Supplementary Table 4** Association between Immune risk score and clinicopathological features of spinal chordoma patients in the validation cohort (n = 60)^a^

| Clinicopathological factors | No. of patients (n = 60) | Immune risk score | | | Test statistic | *P-*value |
| --- | --- | --- | --- | --- | --- | --- |
|  |  | Low (%) | High (%) | |  |  |
| Age (years) |  |  | |  |  |  |
| ≤ 50 | 20 | 12 (60%) | | 8 (40%) | 0.035 | 0.851 |
| > 50 | 40 | 25 (62.5%) | | 15 (37.5%) |  |  |
| Sex |  |  | |  |  |  |
| Male | 42 | 25 (59.5%) | | 17 (40.5%) | 0.272 | 0.602 |
| Female | 18 | 12 (66.7%) | | 6 (33.3%) |  |  |
| Tumor size |  |  | |  |  |  |
| ≤ 5 cm | 22 | 13 (59.1%) | | 9 (40.9%) | 0.097 | 0.755 |
| > 5 cm | 38 | 24 (63.2%) | | 14 (36.8%) |  |  |
| Tumor location |  |  | |  |  |  |
| Sacral vertebra | 45 | 26 (57.8%) | | 19 (42.2%) | 1.152 | 0.283 |
| Mobile spine | 15 | 11 (73.3%) | | 4 (26.7%) |  |  |
| Surrounding  muscle invasion |  |  | |  |  |  |
| Yes | 31 | 13 (41.9%) | | 18 (58.1%) | 10.563 | **0.001** |
| No | 29 | 24 (82.8%) | | 5 (17.2%) |  |  |
| Preoperative recurrence |  |  | |  |  |  |
| Yes | 13 | 5 (38.5%) | | 8 (61.5%) | 2.631 | 0.105 |
| No | 47 | 32 (68.1%) | | 15 (31.9%) |  |  |
| Grade |  |  | |  |  |  |
| High | 42 | 23 (54.8%) | | 19 (45.2%) | 2.824 | 0.093 |
| Low | 18 | 14 (77.8%) | | 4 (22.2%) |  |  |
| Enneking staging |  |  | |  |  |  |
| IA + IB + IIA | 33 | 24 (72.7%) | | 9 (27.3%) | 3.795 | 0.051 |
| IIB + III | 27 | 13 (48.1%) | | 14 (51.9%) |  |  |
| Type of resection |  |  | |  |  |  |
| EI | 24 | 11 (45.8%) | | 13 (54.2%) | 4.242 | **0.039** |
| EA | 36 | 26 (72.2%) | | 10 (27.8%) |  |  |
| Tumor hemorrhage |  |  | |  |  |  |
| No | 17 | 12 (70.6%) | | 5 (29.4%) | 0.799 | 0.371 |
| Yes | 43 | 25 (58.1%) | | 18 (41.9%) |  |  |
| Tumor necrosis |  |  | |  |  |  |
| Absent + Mild | 33 | 22 (66.7%) | | 11 (33.3%) | 0.776 | 0.379 |
| Moderate + Severe | 27 | 15 (55.6%) | | 12 (44.4%) |  |  |
| Ki-67 index |  |  | |  |  |  |
| Low | 26 | 19 (73.1%) | | 7 (26.9%) | 2.527 | 0.112 |
| High | 34 | 18 (52.9%) | | 16 (47.1%) |  |  |
| Tumor PD-L1 |  |  | |  |  |  |
| Positive | 40 | 20 (50%) | | 20 (50%) | 6.910 | **0.009** |
| Negative | 20 | 17 (85%) | | 3 (15%) |  |  |
| Lobular pattern growth |  |  | |  |  |  |
| Yes | 25 | 18 (72%) | | 7 (28%) | 1.936 | 0.164 |
| No | 35 | 19 (54.3%) | | 16 (45.7%) |  |  |
| Overall TILs |  |  | |  |  |  |
| Positive | 34 | 18 (52.9%) | | 16 (47.1%) | 2.527 | 0.112 |
| Negative | 26 | 19 (73.1%) | | 7 (26.9%) |  |  |

Bold indicates *P* < 0.05.

EI, Enneking inappropriate; EA, Enneking appropriate; TILs, tumor-infiltrating lymphocytes; PD-L1,

programmed cell death-1 ligand 1; ^a^analyzed by Chi-square test.


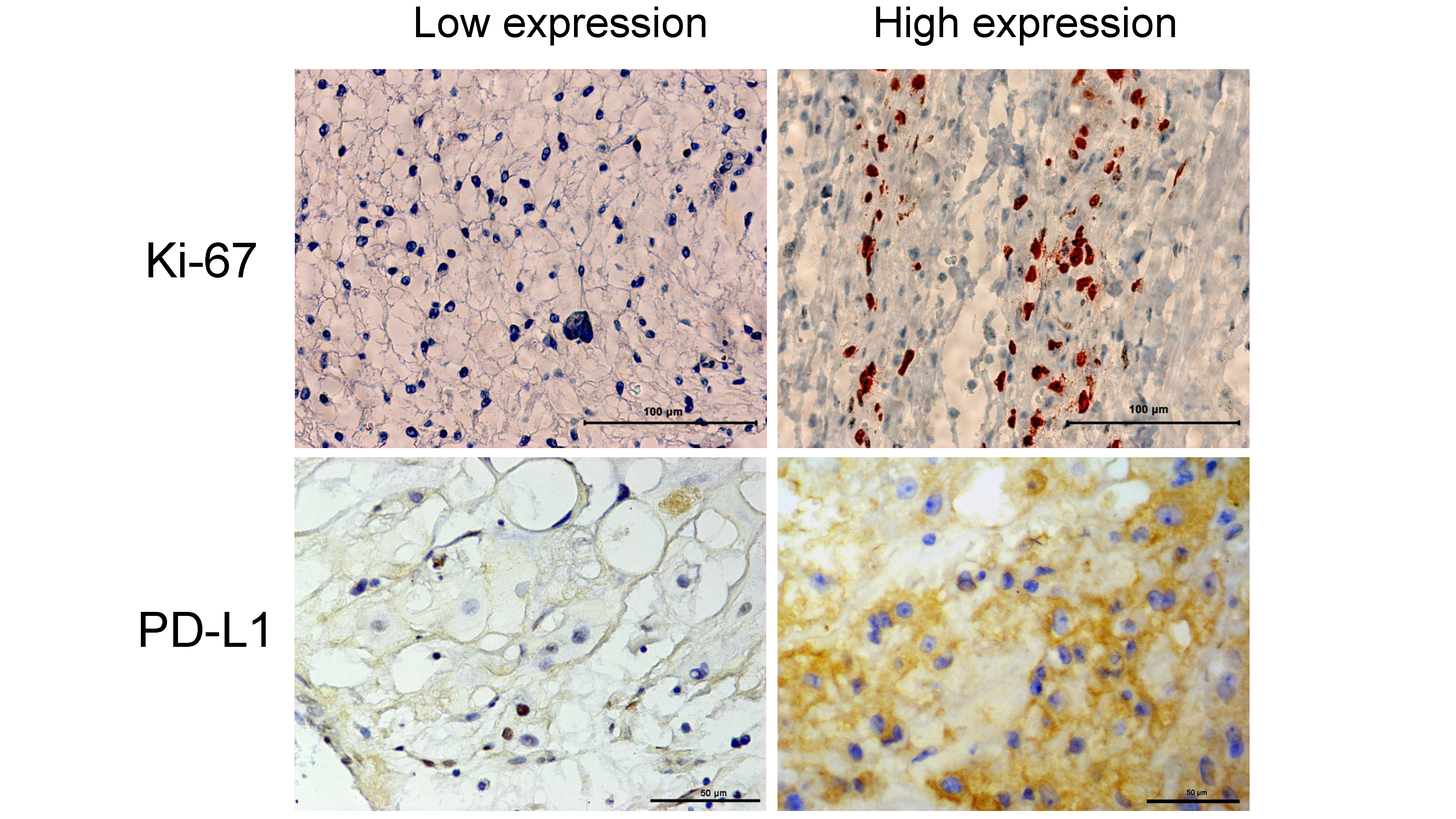


**Supplementary Fig. 1** Representative immunostaining pictures showing high or low expression of Ki-67 index and PD-L1 on chordoma cells (×400).





**Supplementary Fig. 2** A heatmap followed by Pearson uncentered hierarchical clustering (Pearson uncentered algorithm) was represented from R = 1 positive correlation (red) to R = -0.5 negative correlation (blue) in the training (**A**) and validation cohort (**B**). **C** Selection of the tuning parameter λ by ten-time cross-validation in the LASSO model. Solid vertical lines represent partial likelihood deviance ± standard error. The dotted vertical lines are drawn at the optimal value (λ = 1.778E-3 with log λ = -2.750) by minimum criteria. **D** LASSO coefficient profiles of the 10 chordoma-associated immune features. A vertical line is drawn at the value (λ = 1.778E-3 with log λ = -2.750) chosen by 10-fold cross-validation





**Supplementary Fig. 3** Determined cutoff values for immune risk score and densities of tPD-1^+^ TILs, sCD8^+^ TILs, sFoxp3^+^ TILs and tFoxp3^+^ TILs in prognosis analysis with the overall survival as the outcome parameter. PD-1, programmed cell death-1; TILs, tumor-infiltrating lymphocytes; sTILs, stromal TILs; tTILs, tumoral TILs.


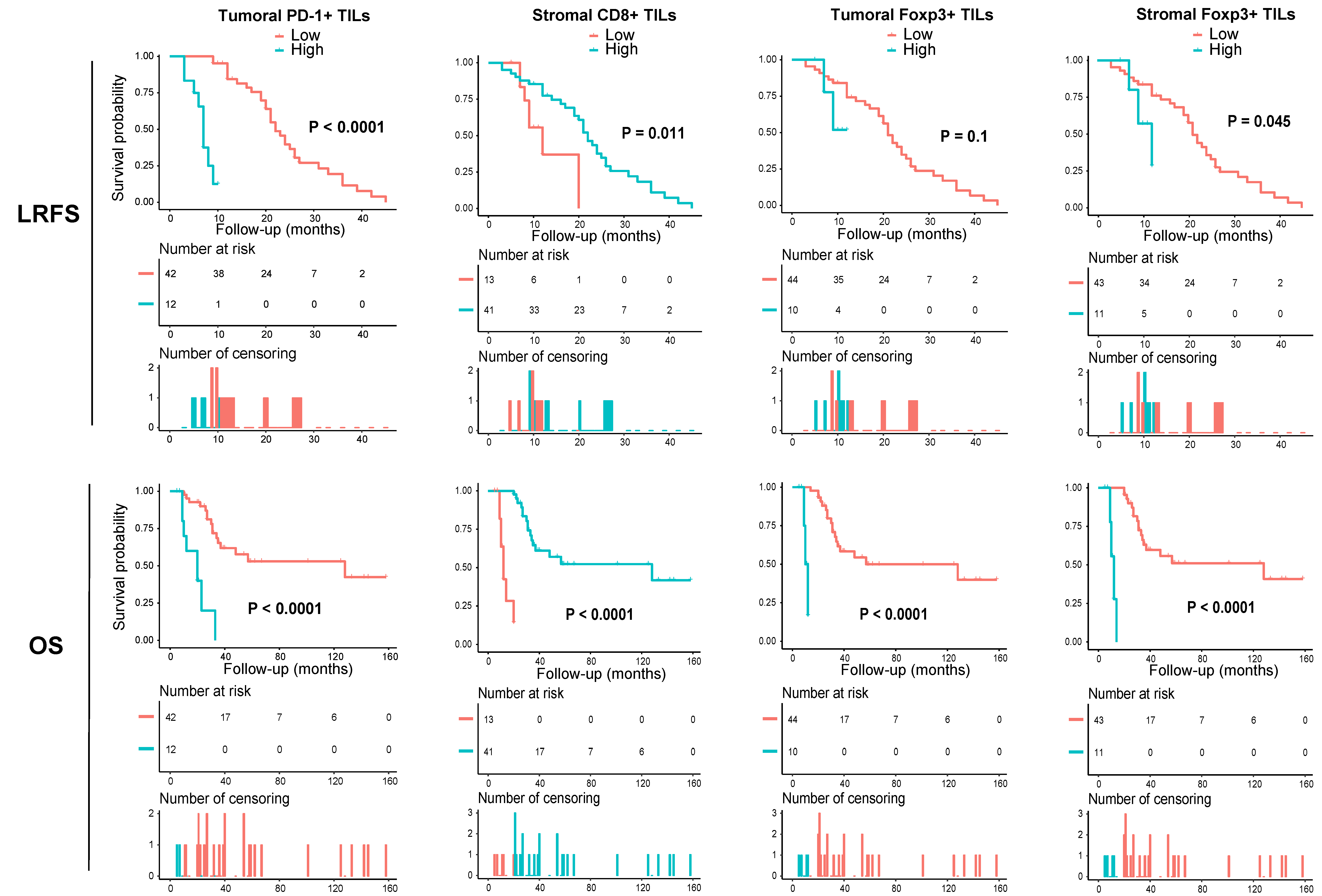


**Supplementary Fig. 4** Kaplan-Meier survival analysis of LRFS and OS for 54 spinal chordoma patients in the training cohort according to the four immune parameters selected for subsequent IRS construction. PD-1, programmed cell death-1; LRFS, local recurrence-free survival; OS, overall survival.


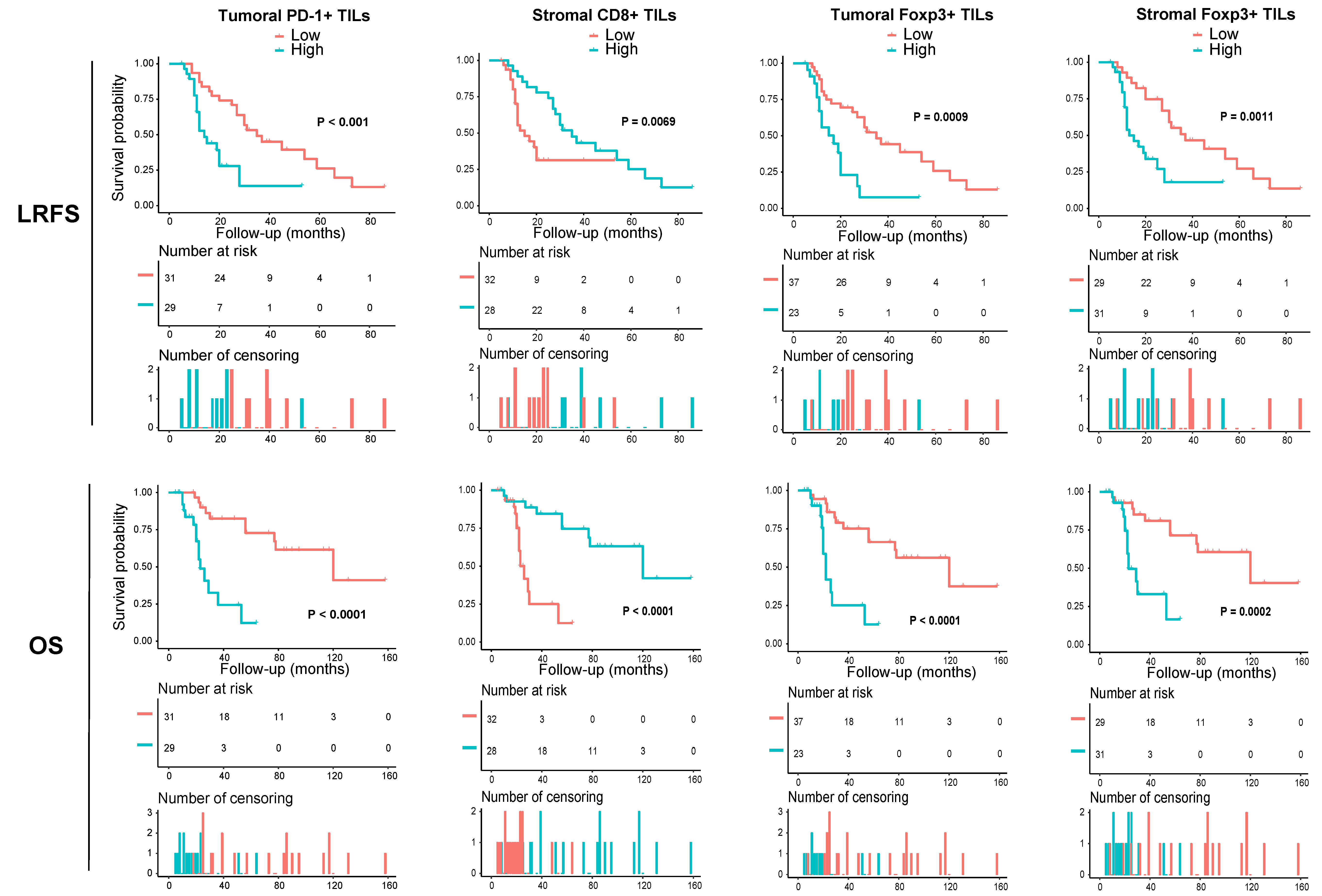


**Supplementary Fig. 5** Kaplan-Meier survival analysis of LRFS and OS for 60 spinal chordoma patients in the validation cohort according to the four immune parameters selected for subsequent IRS construction. PD-1, programmed cell death-1; LRFS, local recurrence-free survival; OS, overall survival.


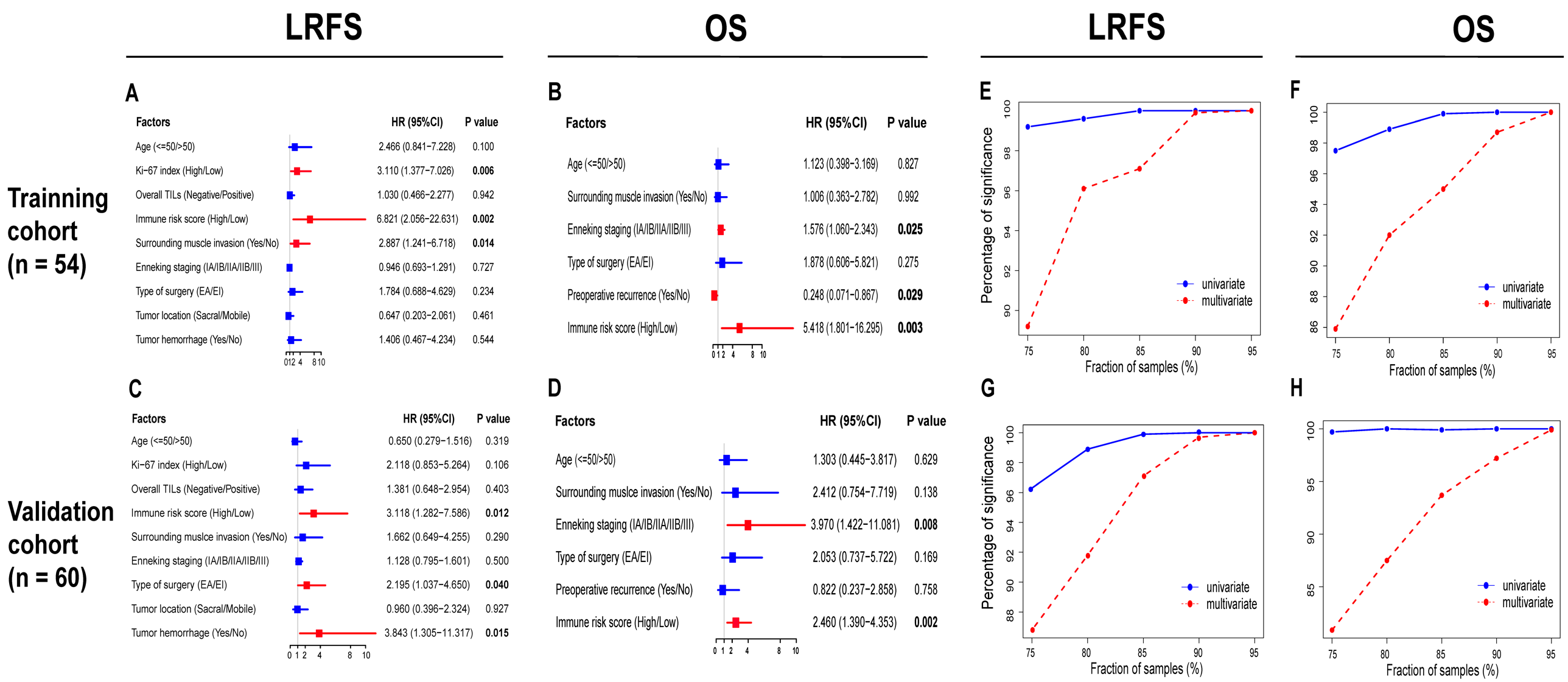


**Supplementary Fig. 6** Multivariate Cox regression model for LRFS amd OS of spinal chordoma patients in the training (**A, B**) and validation (**C, D**) cohort. The boxes indicate the hazard ratio, and the horizontal lines represent 95% confidence intervals. Percentage of times where IRS was statistically significant in univariate and multivariate analysis of LRFS and OS in the training (**E, F**) and validation (**G, H**) cohort with different amounts of patient samples selected randomly (1000 times). IRS, immune risk score; LRFS, local recurrence-free survival; OS, overall survival.


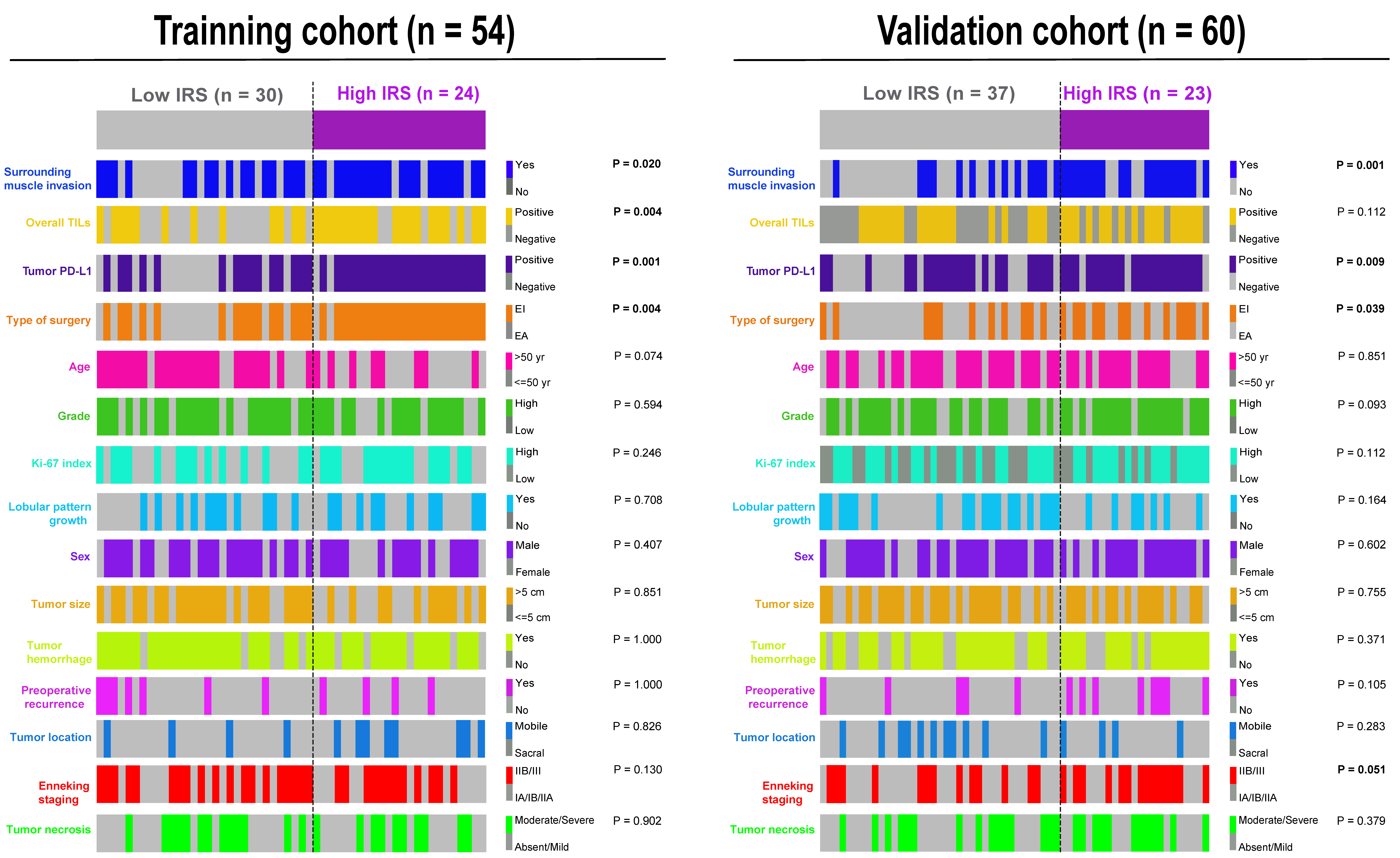


**Supplementary Fig. 7** Heatmap shows the correlation between the IRS and clinicopathological characteristics of spinal chordoma patients in the training (*left*) and validation (*right*) cohort. Columns represent patients who were stratified by the IRS. Black dotted line represents the IRS cutoff dividing patients into high and low subgroups. IRS, immune risk score; EI, Enneking inappropriate; EA, Enneking appropriate.


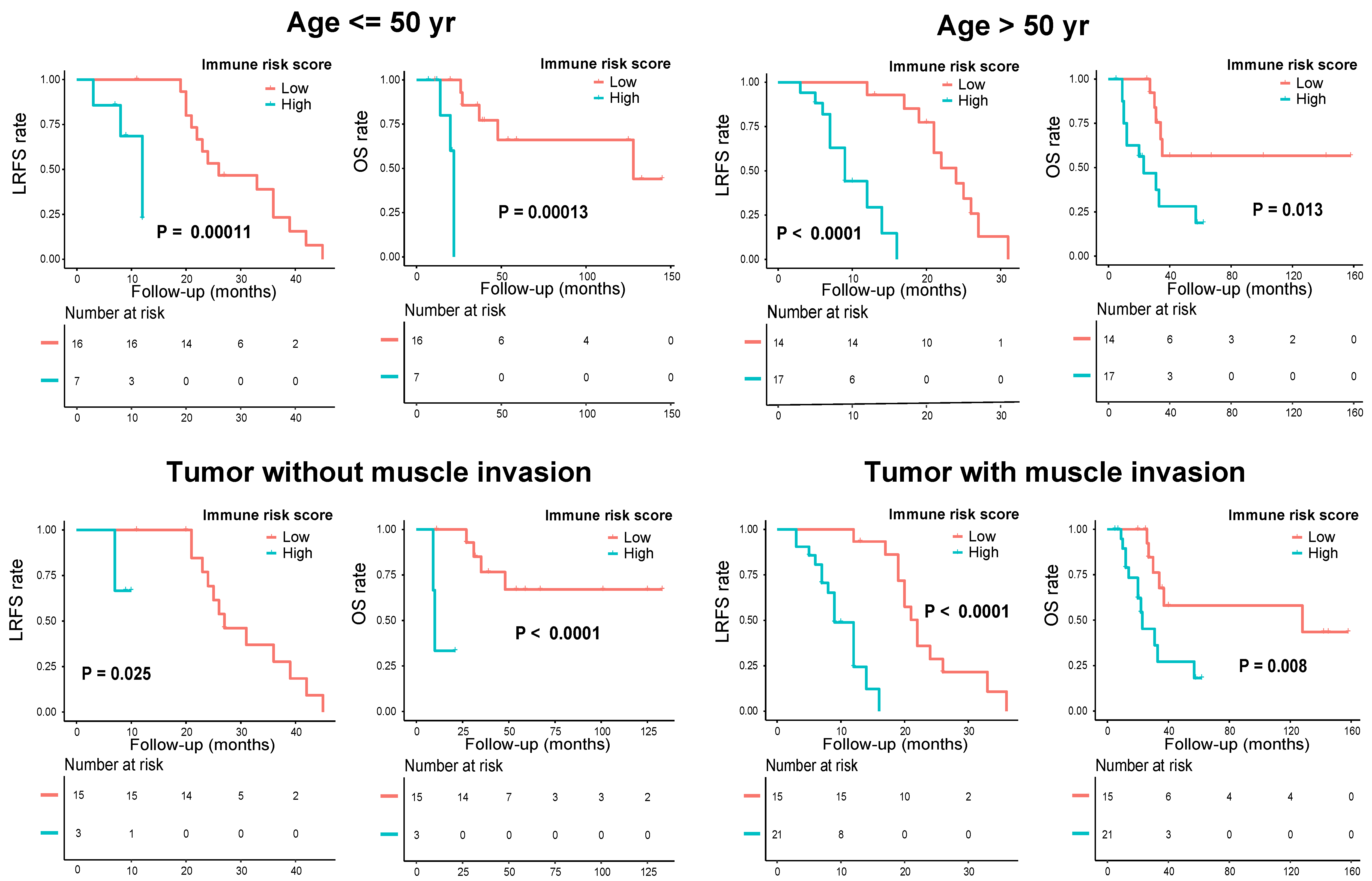


**Supplementary Fig. 8** Kaplan-Meier survival analysis of LRFS and OS for 54 spinal chordoma patients in the training cohort according to the IRS classifier (high or low) stratified by age and tumor invading into surrouding muscle tissues or not. IRS, immune risk score; LRFS, local recurrence-free survival; OS, overall survival.


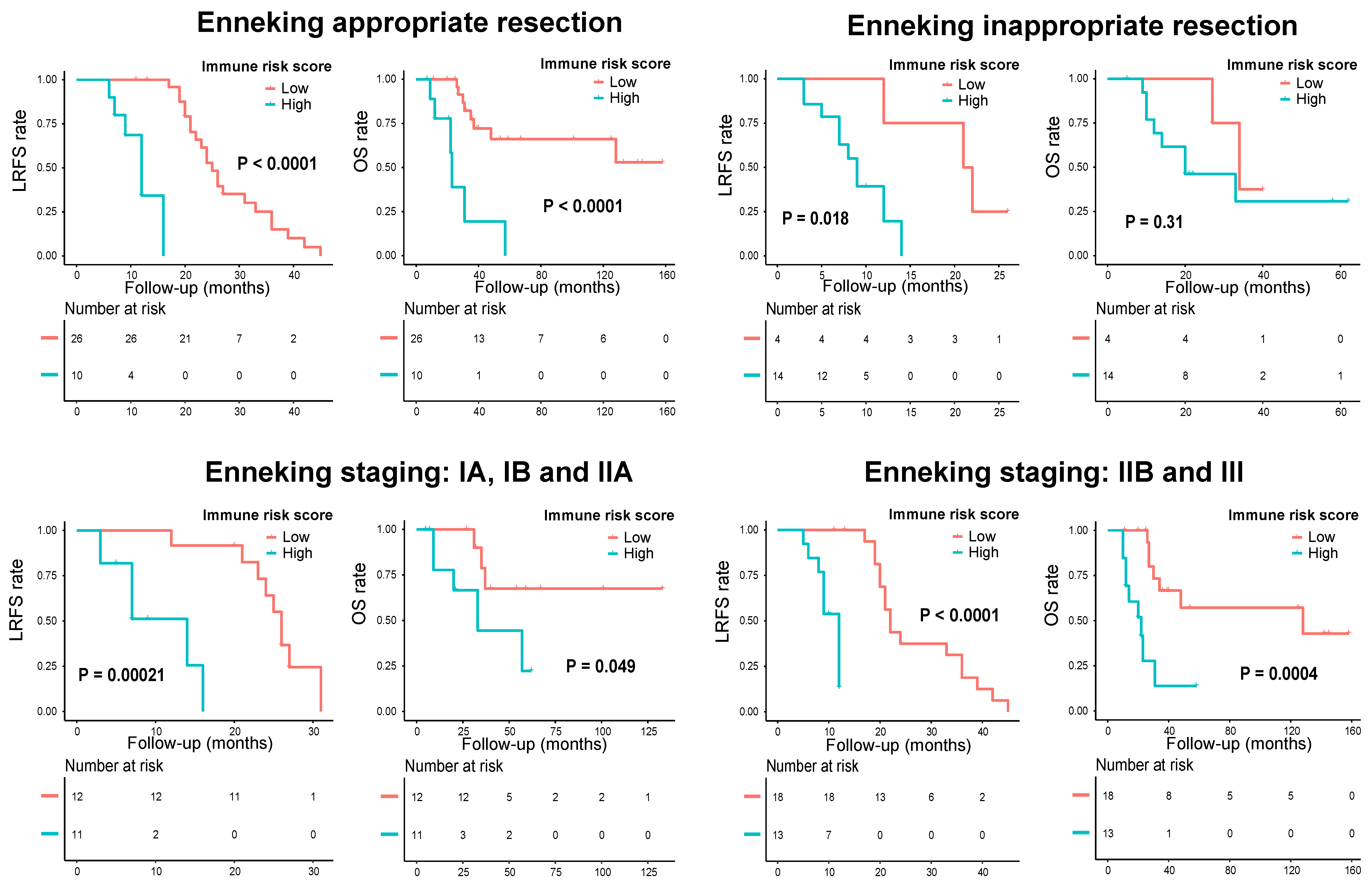


**Supplementary Fig. 9** Kaplan-Meier survival analysis of LRFS and OS for 54 spinal chordoma patients in the training cohort according to the IRS classifier (high or low) stratified by type of surgery and Enneking staging system. IRS, immune risk score; LRFS, local recurrence-free survival; OS, overall survival.


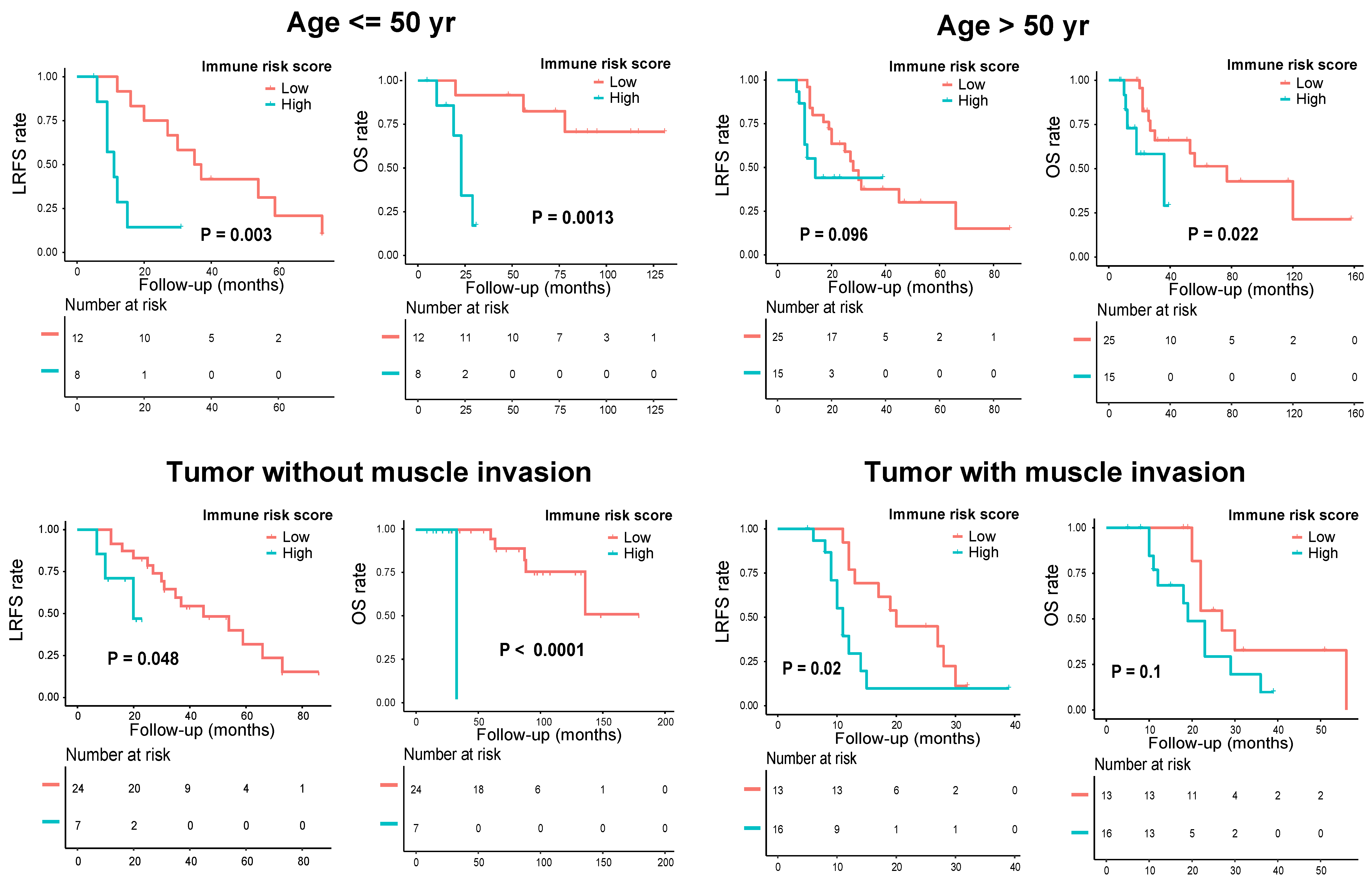


**Supplementary Fig. 10** Kaplan-Meier survival analysis of LRFS and OS for 60 spinal chordoma patients in the validation cohort according to the IRS classifier (high or low) stratified by age and tumor invading into surrouding muscle tissues or not. IRS, immune risk score; LRFS, local recurrence-free survival; OS, overall survival.


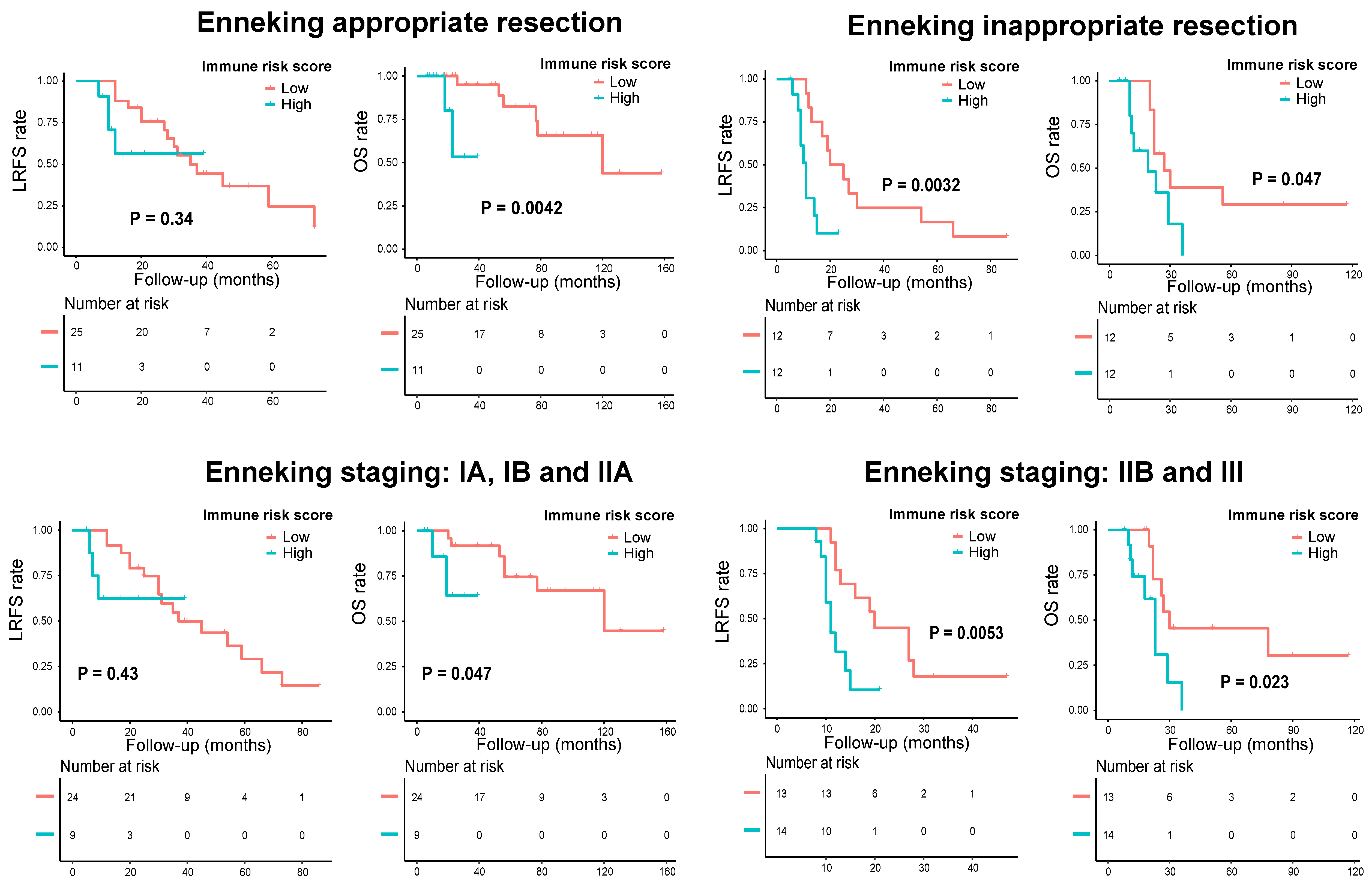


**Supplementary Fig. 11** Kaplan-Meier survival analysis of LRFS and OS for 60 spinal chordoma patients in the validation cohort according to the IRS classifier (high or low) stratified by type of surgery and Enneking staging system. IRS, immune risk score; LRFS, local recurrence-free survival; OS, overall survival.





**Supplementary Fig. 12** Comparison of the prognostic performance by receiver operating characteristic analysis between the IRS classifier and four immune variables for LRFS and OS prediction in the training (**A, B**) and validation (**C, D**) cohort. Comparison of the sensitivity and specificity by receiver operating characteristic analysis between the IRS classifier and significant clinicopathological factors for LRFS and OS prediction in the training (**E, F**) and validation (**G, H**) cohort. The clinical predictors were selected based on literature reports and the results from multivariable Cox analysis, depending on their significant association with LRFS or OS of patients, respectively. IRS, immune risk score; LRFS, local recurrence-free survival; OS, overall survival.
